# Facile Synthetic Access Towards Sulfur- and Selenium-Functionalized Boron-Based Multiresonance TADF Emitters

**DOI:** 10.3390/molecules29245819

**Published:** 2024-12-10

**Authors:** Zeynep Güven, Hadi Dolati, Leo Wessel, René Frank

**Affiliations:** Department of Inorganic and Analytical Chemistry, Technische Universität Braunschweig, Hagenring 30, 38106 Braunschweig, Germany; z.gueven@tu-bs.de (Z.G.); h.dolati@tu-bs.de (H.D.); leo.wessel@tu-bs.de (L.W.)

**Keywords:** thermally activated delayed fluorescence (TADF), multiple resonance (MR), triarylborane, narrowband emission, spin–orbit coupling, heavy atom effect, sulfur, selenium, helicity, OLED

## Abstract

Thermally activated delayed fluorescence (TADF) materials with high photoluminescence quantum yields and a fast reverse intersystem crossing (RISC) are of the highest interest for organic light-emitting diodes (OLEDs). In the past decade, triaryl boranes with multiple resonance effect (MR) have captured significant attention. The efficiency of MR-TADF emitters strongly depends on small singlet–triplet energy gaps (ΔE_ST_), but also on large reverse intersystem crossing (RISC) rate constants (k_RISC_). The latter effect has strongly been focused on very recently and has drawn attention to heavier elements, including sulfur and selenium, the large spin–orbit coupling (SOC) of which accelerates RISC effects. Within the context of MR-TADF emitters, the 5,9-X_2_-13b-boranaphtho [3,2,1-de]anthracene scaffold (X-B-X, X = donor heteroatom, e.g., N, O, S, Se) has been recognized as a promising narrowband-emissive TADF material. However, the incorporation of sulfur and selenium as highly SOC-inducing elements has proven to be difficult. Most synthetic strategies apply protocols initially suggested by Hatakeyama to obtain nitrogen- and oxygen-doped materials. We present an alternative route over the established methodology, which affords highly sought-after sulfur- and selenium-doped materials with a high yield and purity.

## 1. Introduction

The early invention of organic light-emitting diodes (OLEDs) in 1987 has stimulated interest in the design strategies of the active luminophore due to their enormous industrial and commercial application in lighting and displays [1]. Compared to traditional light sources, including hot wire bulbs, discharge lamps or inorganic light-emitting diodes, OLEDs offer high efficiency, ultrathin thickness and high color purity as superior advantages, which can be controlled by the tailor-made design of the active emissive organic luminophore [2,3,4,5,6,7].

**Scheme 1:**
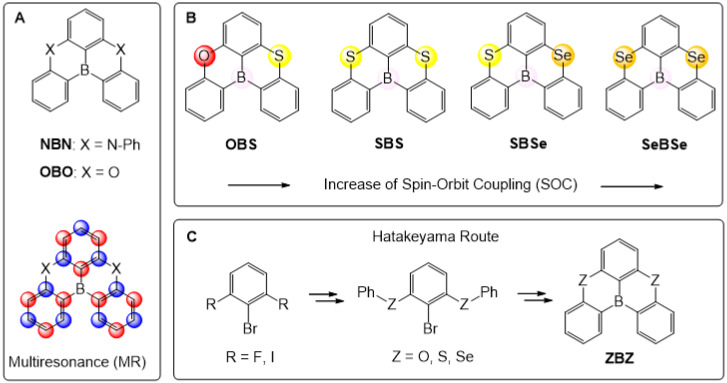
(**A**) The prototypical compounds **OBO** and **NBN** with inherent multiresonance (MR) effect. Blue and red regions represent electron rich and electron deficient *p_z_*-orbitals, respectively. (**B**) The incorporation of heavier chalcogens sulfur and selenium gives rise to electronic perturbation by Spin–Orbit coupling (SOC). (**C**) The synthetic route developed by Hatakeyama has been employed as a standard access towards **ZBZ**-type compounds.

An efficient harvesting process of electrical energy applied in an OLED device deploys the spin statistics of 25% singlet and 75% triplet excitons. An ideal luminophore traps both types of excitons and gives rise to first excited singlet S_1_ and triplet T_1_ energy levels. While direct radiative emission from S_1_ to the ground state S_0_ is usually rapid at a rate constant of 10^6^ s^−1^, the emission from the triplet state T_1_ can proceed with interconversion to the singlet state S_1_ by way of the reverse intersystem crossing (RISC). The latter process is spin-forbidden, and the limiting step has a usual rate of 10^4^ s^−1^ for purely organic luminophores. However, a low singlet–triplet gap (ΔE_ST_) between the S_1_ and T_1_ energy levels allows for the thermally driven RISC, which gives rise to the well-known thermally activated delayed fluorescence (TADF) effect. Besides the low singlet–triplet gap, the participation of heavier elements, which introduce spin–orbit coupling (SOC) between S_1_ and T_1_, can additionally contribute to RISC in purely organic TADF emitters to obtain rate constants of up to 10^8^ s^−1^ [8,9,10,11]. From the standpoint of practical luminophore design the element boron has attracted much attention [12,13,14,15,16]. In conventional boron-based TADF emitters, the boron entity was exploited to rigidify the organic aromatic scaffold, the latter of which contained electronically separated donor (D) and acceptor (A) units. The harvesting mechanism was purely based on an intrinsic intramolecular charge transfer (ICT) from donor to acceptor entities (D → A) without any photophysical contribution of the boron atom itself. However, this D–A-type TADF emission inevitably causes substantial structural relaxation between the ground and excited states, resulting in the broadening of the emission spectrum with a large full width at half maximum (FWHM ≥ 70 nm). Such broad emissions reduce the resulting color purity of organic light-emitting diodes. A recently introduced concept by Hatakeyama et al. is based on the multiresonance (MR) effect of triaryl boranes, in which the alternating sequence of HOMO and LUMO orbitals in the aromatic scaffold can be controlled by the 1,2-positions of a Lewis acidic boron atom and Lewis basic nitrogen or oxygen sites, Scheme 1A [17,18,19]. Due to the opposite resonance effects of boron (−*M*) and nitrogen or oxygen (+*M*), the pendant arene adopts a sharply alternating sequence of electron donating or accepting carbon *p_z_*-orbitals. In the prototypical compounds **NBN** and **OBO** the atomic alternation of HOMO and LUMO orbitals over the rigid aromatic scaffolds suppresses structural relaxation and vibronic coupling, leading to small singlet–triplet gaps (ΔE_ST_ = 0.10–0.20 eV), narrowband emissions and high photoluminescence quantum yields.

While most of the early MR-TADF materials display low RISC rates in the order of 10^4^ s^–1^, the incorporation of heavier elements (sulfur, selenium) has been demonstrated to significantly accelerate the RISC process in MR-TADF emitters based on the inherent spin–orbit coupling (SOC). However, reports on boron-based MR-TADF materials with enhanced SOC are still rare due to the very recent origin of this area. Materials with different donor atoms, i.e., combinations of nitrogen and sulfur or selenium, displayed enhanced RISC rate constants between 10^6^ s^−1^ and 10^8^ s^−1^ resulting in excellent electroluminescence quantum efficiency values of ~28% [20,21,22,23]. A systematic replacement of sulfur at the oxygen position in compound **OBO** gave rise to compounds **OBS** and **SBS** as displayed in Scheme 1B. In the order of rising sulfur content the singlet–triplet gaps (ΔE_ST_) decreased, and in combination with enhanced spin–orbit coupling (SOC), the RISC rate was found to be up to ~10^5^ s^−1^ [24]. Only very recently, the compound **SeBSe** was published, which gave the lowest singlet–triplet gap of ΔE_ST_ = 0.13 eV and the highest RISC rate value of ~10^8^ s^−1^ in this series as shown in Scheme 1B [25]. According to theoretical and computational studies, the systematic chalcogen variation is a highly efficient and versatile tool used to control the photophysical properties in MR-TADF systems [26,27]. Recently, various derivatives of the parent SBS scaffold were prepared and the subjected to intense photophysical investigation [28,29,30,31].

## 2. Results and Discussion

**Scheme 2:**
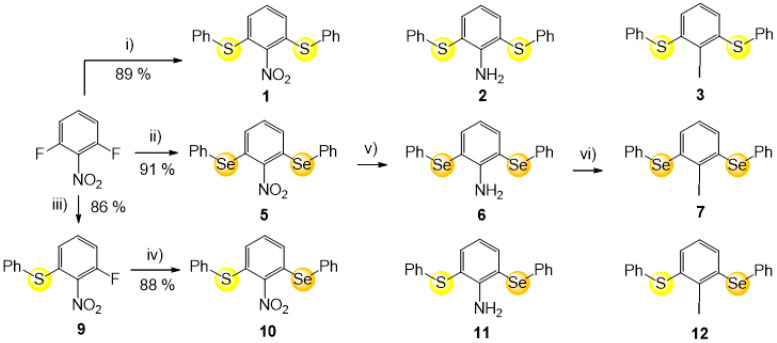
Synthetic access to iodo precursors **3**, **7** and **12**. Reagents and conditions: (i) 2 eq. PhSH, K_2_CO_3_, DMF, rt, 16 h. (ii) 2 eq. NaSePh, DMF, 100 °C, 16 h. (iii) 0.5 eq. PhSH, K_2_CO_3_, DMF, rt, 16 h. (iv) 1 eq. NaSePh, DMF, 100 °C, 16 h. (v) Zn, NH_4_Cl, MeOH, 65 °C, 0.5–2 h, ≥90%. (vi) NaNO_2_, HCl, MeCN, H_2_O, −10 °C, then KI, 60 °C, 1 h, ≥90%.

These promising outcomes have stimulated interest in **ZBZ**-type compounds as highly sought-after efficient exciton harvesting materials with narrowband emission. However, the synthetic routes towards these materials are currently limited. A route reported by Hatakeyama was deployed as the standard approach for these materials [12], in which halogenated benzene derivatives 1,3-R_2_-2-BrC_6_H_3_ are functionalized to give the intermediates 1,3-(PhZ)_2_-2-BrC_6_H_3_ shown in Scheme 1C, step (i). The sequential lithium bromine exchange with *n*-BuLi followed by the addition of boron tribromide in the presence of amine bases ultimately affords the heteroaromatic final products **ZBZ** as displayed in Scheme 1C, step (ii). While this approach gave compounds **OBS** and **SBS** in ca. 20% yield, compound **SeBSe** was more difficult to be obtained with an outcome of only 9% in the final step of the synthetic protocol. Moreover, to the best of our knowledge, compound **SBSe** has not been obtained yet and is a missing member of this series. In view of the importance of these compounds and due to the currently low yields obtained in Hatakeyama’s route, we set out to develop an improved strategy, Scheme 2. With the focus on compounds **SBS**, **SBSe** and **SeBSe**, we assumed that the C–Br bonds in the intermediates 1,3-(PhZ)_2_-2-BrC_6_H_3_ exhibit poor reactivity in lithium bromide exchange reactions with alkyl lithium reagents. In particular, the bond dissociation energy (BDE) of C–Br bonds (285 kJ/mol) vs. C–Se bonds (245 kJ/mol) indicate a weaker character for the latter [32]. This fact is in line with reports that various lithium alkyl reagents can undergo facile scission of C–Se bonds in aromatic selenoethers (in THF even at −78 °C) according to Ar_2_Se + LiR → ArSeR + LiAr [33,34,35,36,37], which would also account for the very low yield (9%) of compound **SeBSe**. Our improved methodology therefore involves iodo instead of bromo substituents for the lithium exchange protocol. Since the bond dissociation energy of C–I bonds (215 kJ/mol) is considerably lower than that of C–Br and C–Se bonds [38], and due to the fact that lithium halide exchange mechanisms are much more facile for iodoarenes compared to bromoarenes [39], we formulated compounds **3**, **7** and **12** as suitable precursors for the formation of the target boron heterocycles **ZBZ**.

**Scheme 3:**
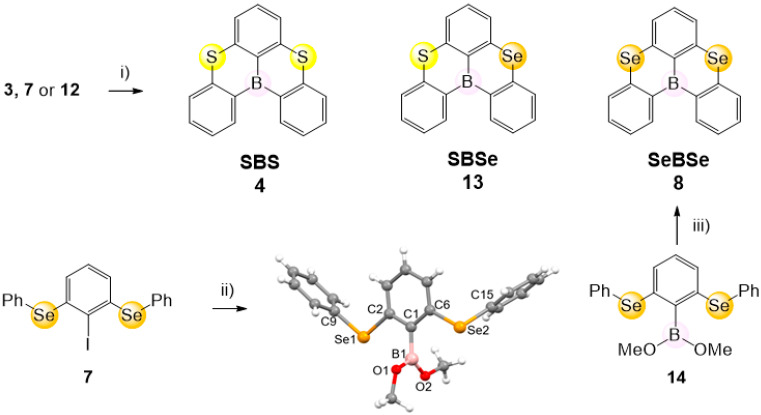
Synthetic access towards boronated MR-TADF **ZBZ**-type emitters **4**, **8** and **13**. Reagents and conditions: (i) 1.05 eq. *n*-BuLi, *m*-xylene, −30 °C, 1 h, then 50 °C, then −30 °C, 1.20 eq. BBr_3_, 2.50 eq. NEt(*i*Pr)_2_, 125 °C, 12 h, 81% (for **4**), 67% (for **8**) and 75% (for **13**). (ii) 1 eq. *n*-BuLi, *m*-xylene, −30 °C, 1 h, then 50 °C, then −30 °C, B(OMe)_3_, rt, 1 h. (iii) 2.20 eq. BBr_3_, *m*-xylene, rt, 1 h, then 3.00 eq. NEt(*i*Pr)_2_, 125 °C, 12 h.

In view of the advantages associated with iodoarenes in lithium halide exchange reactions, we were surprised that this compound class was rarely used in the synthesis of MR-TADF emitters. Iodoarenes started to be employed in the synthesis of multiresonant boron/nitrogen/oxygen-based emitters only recently [40,41]. In our approach 1,3-difluoro-2-nitrobenzene was employed as a suitable starting material to introduce the phenylthio or phenylseleno moieties in compounds **1**, **5**, **9** and **10**. The combination of fluoro substituents as efficient leaving groups in aromatic systems and the nitro group with assisting the *+M*-effect renders the starting material highly susceptible in nucleophilic substitution reactions with satisfying yields in all steps. Reactions towards phenylthiol-ethers **1** and **9** [steps (i) and (iii)] were facile at ambient temperature in dimethylformamide (DMF), while the introduction of phenylseleno groups in compounds **5** and **10** [steps (ii) and (iv)] required heating to 100 °C in the same solvent. This observation is in line with the stronger nucleophilicity of PhS^−^ vs. PhSe^−^ anions in aprotic solvents, in this case DMF. The reduction of the nitro group in compounds **1**, **5** and **10** was conveniently performed with zinc and ammonium chloride as a mild acid in methanol. Interestingly, the selenium derivatives **6** and **11** were formed much faster than the fully sulfurated compound **2** with full conversion observed between 30 min and 2 h. The amino derivatives **2**, **6** and **11** were converted towards the target iodo arenes **3**, **7** and **12** in a Sandmeyer-type reaction via intermediate diazonium salts in excellent yields of ≥90%. All compounds **1**–**12** were obtained analytically pure and were full characterized by ^1^H and ^13^C NMR spectroscopy. Unexpectedly, compounds **3**, **7** and **12** showed very limited solubility in organic solvents, including chloroform and dichloromethane. Single crystals suitable for X-ray crystallography were obtained for iodo arenes **3**, **7** and **12**. The analysis confirmed the chemical identity within the space group *P*2_1_/c and similar packing patterns in all three cases as shown in Figures S5, S15 and S29. The inspection of the molecular packing did not disclose any unusually strong interactions or short distances, which would account for the poor solubility of **3**, **7** and **12**, and the origin of such behavior remains currently unclear. With iodoarenes **3**, **7** and **12** at hand, the cyclization towards triarylboranes of type **ZBZ** was performed as displayed in Scheme 3. In all three cases, the lithium iodine exchange with *n*-BuLi was found to proceed in a facile way at −30 °C in *m*-xylene followed by the addition of boron tribromide.

**Scheme 4:**
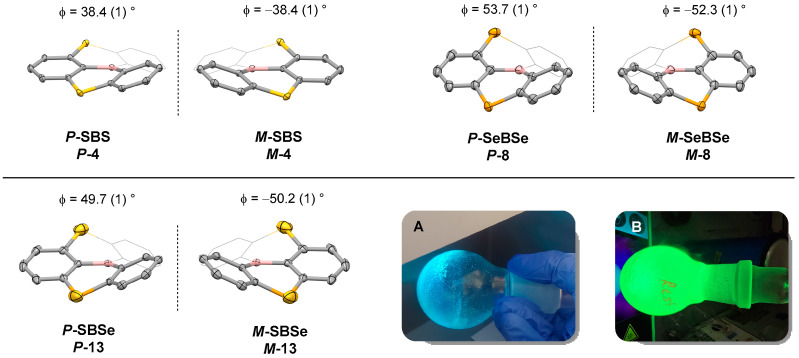
Molecular structures of compounds **ZBZ** (**4**, **8** and **13**) obtained from X-ray crystallographic analysis. Helical isomers are displayed in *P*- and *M*-configuration with mathematically positive (*P*) or negative (*M*) helical orientation. Compound **13** (solid) with 365 nm excitation (not de-aerated): Insert A—at ambient temperature. Insert B—at 77 K. The angle Φ denotes the torsion angle between the terminal *o*-phenylene groups.

The cyclization reaction occurred with heating in the presence of Hünig base and afforded compounds **4** (**SBS**), **13** (**SBSe**) and **8** (**SeBSe**) in a yield of 81%, 75% and 67%, respectively. While compound **13** was not reported, the yields of **4** and **8** are highly satisfying, particularly in view of the low yield reported earlier for compound **8** (9%). All three compounds were obtained as crystalline yellowish materials in analytically pure quality and were fully characterized by NMR spectroscopy. The ^11^B{^1^H} NMR spectra show broad singlets, which appear in the diagnostic region for triarylboranes and display low-field shift in the order of **4** (46.6 ppm), **13** (48.2 ppm) and **8** (52.3 ppm). The excellent yields for compounds **4**, **8** and **13** confirm the initial hypothesis that iodo arenes provide significant benefits over bromoarenes. We assume that the metal halide exchange to produce the intermediate lithium aryl is more facile for iodoarenes. Although Hatakeyama’s route is a very complex procedure, which involves lithium halide exchange followed by the formation of aryl dibromoborane and the cyclization reaction, we were surprised at the absence of any reports to isolate intermediates from this procedure. Therefore, we wish to contribute insight into the formation of intermediates in our protocol, and focused on the most sensitive iodoarene **7** from our series, Scheme 3. The elaborate lithium iodine exchange followed by the addition of trimethyl borate [B(OMe)_3_] affords the aryl boronic dimethyl ester **14** in a yield of 94% as shown in Scheme 3, step (ii). Compound **14** can be considered as being formed from the intermediate aryllithium in the sequence 1,3-(PhSe)_2_-2-**I**C_6_H_3_ (**7**) → 1,3-(PhSe)_2_-2-LiC_6_H_3_ → 1,3-(PhSe)_2_-2-**(MeO)_2_B**C_6_H_3_ (**14**). We interpret the formation of compound **14** in a high yield as a proof of the intermediacy of aryl lithium 1,3-(PhSe)_2_-2-LiC_6_H_3_, which then afforded the isolated compound **14**. The latter was obtained analytically pure and was fully characterized by NMR spectroscopy. The ^11^B{^1^H} NMR spectrum displays a broad singlet at 28.0 ppm, which appears in the diagnostic region for boronic esters. The X-ray crystallographic analysis of compound **14** confirmed the identity as a boronic ester, with the B(OMe)_2_ group in almost perpendicular alignment to the central aryl group, as shown in Scheme 3 and Figure S37. In further experiments, compound **14** was treated with boron tribromide and Hünig base as displayed in Scheme 3, step (iii). With the same conditions applied above, compound **SeBSe** (**8**) as a cyclic product was formed in a yield of 70%, which shows that compounds of type **14** (with bromo substituents) may operate as intermediates. Crystals suitable for X-ray crystallographic analysis could be obtained by slow solvent evaporation of benzene solutions for the series of compounds **4**, **8** and **13**, as shown in Scheme 4 and Figures S8, S18 and S32. The analysis confirmed the structural identity of all compounds.

Although crystal structures of compounds **4** [24] and **8** [25] were reported earlier, we found deviations from the previous metric data exceeding the crystallographic errors and wish to publish our data together with those of the new compound **13**. All compounds in this series display a significant distortion from planarity, which could be expected for (hetero)aromatic polycycles. The out-of-plane bending can be traced back to (i) longer bond distances of C–S and C–Se bonds over the C–C bonds of the aromatic scaffold, and (ii) the contraction of the bond angles C–S–C and C–Se–C over the close to 120° C–C–C bond angles in planarized aromatic structures. Due to the distortion, compounds **4**, **8** and **13** give rise to helical chirality, according to which *P*- or *M*-enantiomers can be formed depending on the mutual orientation of the terminal *o*-phenylene entities. We measured the angle of inclination Φ between the *o*-phenylene units, where positive or negative angles indicate *P*- or *M*-enantiomers, respectively. Compound **4** crystallized in the space group *Pbca* with one independent molecule of **4** in the asymmetric unit. The presence of three types of glide planes in this space group generates the mutual *P*- or *M*-enantiomers by symmetry and gives rise to equal angles of Φ by magnitude. In contrast, compounds **8** and **13** both crystallized in space groups *P*2_1_2_1_2_1_ with two independent molecules of *P*- and *M*-configuration. The absence of symmetry relations gives rise to unequal angles Φ by magnitude within the crystallographic error for *P* and *M*-enantiomer. In compounds **4**, **8** and **13**, the distortion is found to rise according to the stated order: **4** [Φ = +/− 38.4 (1) °] → **13** [Φ = +49.7(1) and −50.2(1)°] → **8** [Φ = +53.7(1) and −52.3(1)°], which can be rationalized with the rising incorporation of the heavier chalcogen selenium. Since this work focusses on the improved synthetic access and the structural characterization of the sulfur- or selenium-substituted ZBZ-type borane, we wish to postpone a comparative photophysical characterization of the compounds, including the production of OLED devices, to a later publication. However, preliminary exposure of a solid sample of compound **13** to a source of radiation at 365 nm revealed a blue emission at ambient temperature, which shifted to an intense, green emission upon cooling to 77 K as displayed in Scheme 4 and inserts A and B. This behavior is a strong indication for TADF-based emission with significant spin–orbit coupling (SOC), according to which the fluorescence occurs from the slightly higher lying S_1_ state, which is thermally populated at ambient temperature, while cooling populates the lower lying T_1_ triplet state with phosphorescence emission.

## 3. Conclusions

Multiresonant triaryl boranes are an important class of TADF emitters in the production of OLED devices. The incorporation of Lewis basic donor atoms in conjugation with Lewis acidic boron sites is essential to give atomically separated HOMOs and LUMOs with low singlet–triplet gaps (ΔE_ST_). The incorporation of heavier donor centers, i.e., sulfur or selenium, is expected to enhance TADF effects with an increase of the reverse intersystem crossing (RISC) due to the elevated spin–orbit coupling of these elements. However, the synthesis of these **ZBZ**-type boranes via routes developed by Hatakeyama suffered from extremely low yields. Our elaborate protocol demonstrates that the inherent weakness of the C–I bond in iodoarenes is essential in the lithiation step and produces established (**4**, **8**) and new (**13**) **ZBZ**-type boranes in satisfying yield and purity [42,43,44,45,46,47,48,49,50,51]. We present evidence for the lithiated intermediates in a trapping reaction to afford the respective boronic dimethylester **14**, which is the first time that a boron-containing intermediate could be isolated in this complex procedure. Structural elucidation disclosed helical chirality for compounds **4**, **8** and **13**. Preliminary experiments indicated temperature dependent fluorescent and phosphorescent emission, which will be studied in depth, including the fabrication of OLED devices, in the future.

## Data Availability

Experimental procedures and full data of characterization including images of NMR spectra can be found in the file Supplementary Materials. Crystallographic information files (cif) have been deposited under the respective CCDC numbers 2394813 (**3**), 2394817 (**4**), 2394812 (**7**), 2394816 (**8**), 2394814 (**12**), 2394815 (**13**), 2394811 (**14**) and can be downloaded free of charge at https://www.ccdc.cam.ac.uk (accessed on 9 December 2024).

## Supplementary Materials

The following supporting information can be downloaded at: https://www.mdpi.com/article/10.3390/molecules29245819/s1, References [52,53,54] are cited in Supplementary Materials file.
