# Supplementary material for: Facile Synthetic Access Towards Sulfur- and Selenium-Functionalized Boron-Based Multiresonance TADF Emitters
Source: Molecules. 2024 Dec 10;29(24):5819. doi: 10.3390/molecules29245819 (PMC11679814; doi:10.3390/molecules29245819)
Supplement: Supplementary file 1 [file molecules-29-05819-s001.zip › molecules-3339951-supplementary.pdf]

Facile Synthetic Access Towards Sulfur and Selenium Functionalized Boron Based Multiresonance TADF emitters.

Zeynep Güven, Hadi Dolati, Leo Wessel, and René Frank *

Supporting Information

1. Synthetic and Analytical Procedures

1.1 General Information

All procedures were performed in nitrogen atmosphere unless otherwise stated. Commercially available compounds (Abcr, Deutero, Sigma Aldrich, TCI) were used without further purification. NMR spectra were measured using Bruker Avance II-300, Avance III-HD, Avance III-400, and AVII-600 spectrometers. The chemical shifts (δ) are given in parts per million (ppm). As a calibration method ^{13}C resonances of the solvent peaks were used ($CDCl_3$, δ = 77.16 ppm; CD_2Cl_2, δ = 53.84 ppm). For ^{11}B-NMR spectra an external calibration with $BF_3{\cdot}Et_2O$ was used. Coupling constants are stated in Hertz (Hz), multiplicities are defined as br (broad), s (singlet), d (doublet), t (triplet), q (quartet), qu (quintet), sept (septet) or m (multiplet). Elemental analyses were accomplished by combustion and gas chromatographic analysis using a Vario MICRO Tube and HW detection. Values are reported in weight-%.

2. Synthesis of Compounds

2.1 Synthesis of Compound 1

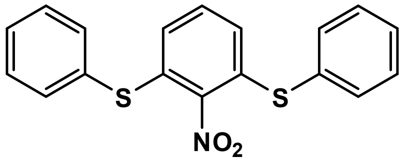

1

Thiophenol (2.00 eq., 2.57 mL, 25.1 mmol) and 1,3-difluoro-2-nitrobenzene (1.00 eq., 2.00 g, 12.6 mmol) were added to a stirred suspension of K_2CO_3 (3.00 eq., 5.20 g, 37.7 mmol) in dry dimethylformamide (DMF, 15 mL) at 0 °C. After stirring for 16 h at 25 °C, dichloromethane (DCM, 100 mL) was added. The organic phases were washed with water and a saturated aqueous solution of $NaHCO_3$ (100 mL each). The organic layer was dried over $MgSO_4$ and filtered. The solvent was removed to obtain compound **1** in a yield of 89 % as a yellow powder.

^{1}H NMR (300 MHz, CD_2Cl_2): δ (ppm) = 7.49 (m, 4H, aryl-CH), 7.40 (m, 6H, aryl-CH), 7.10 (dd, ^3J= 7.45 Hz, 1H, aryl-CH), 6.90 (d, ^3J= 8.0 Hz, 2H, aryl-CH).

^{13}C{^{1}H} NMR (75 MHz, CD_2Cl_2): δ (ppm) = 134.78 (aryl-CH), 134.63 (aryl-C), 134.30 (aryl-CH), 132.30 (aryl-C), 131.32 (aryl-CH), 130.17 (aryl-CH), 129.56 (aryl-CH), 129.25 (aryl-CH).

Elemental analysis: Calculated for $C_{18}H_{13}NO_2S_2$: C, 63.69; H, 3.86; N, 4.13. Found: C, 64.08; H, 3.95; N, 4.16.

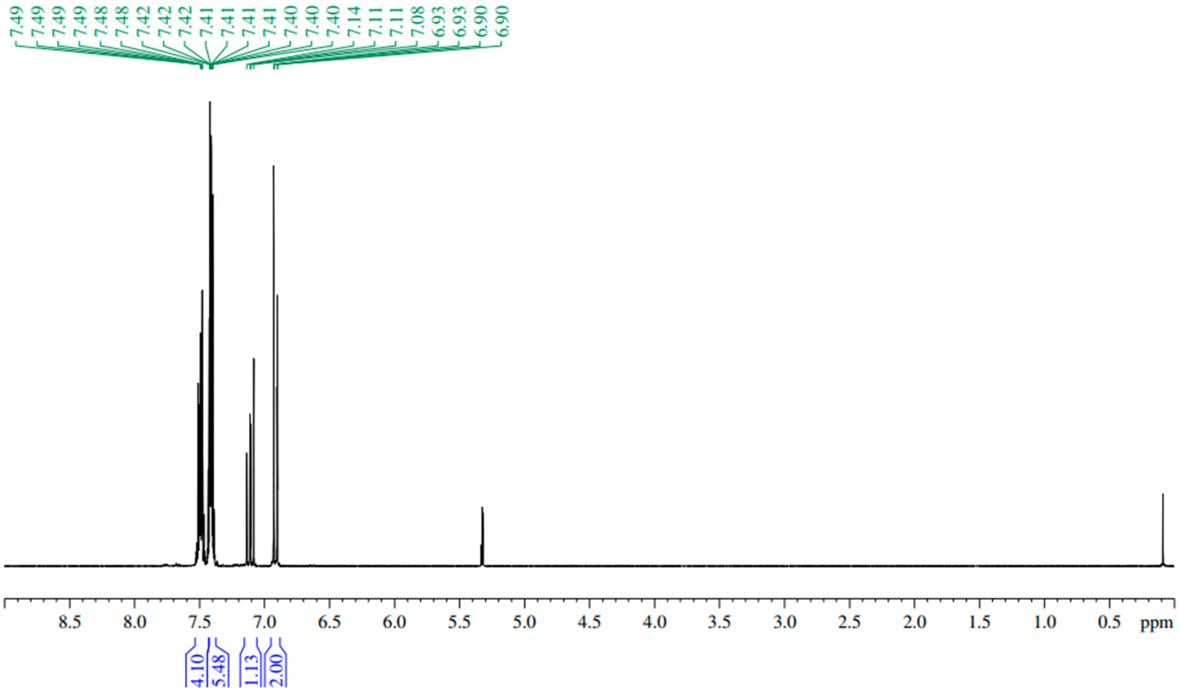

Figure S1: ^{1}H NMR spectrum of compound **1**, CD_2Cl_2, 300 MHz, 25 °C.

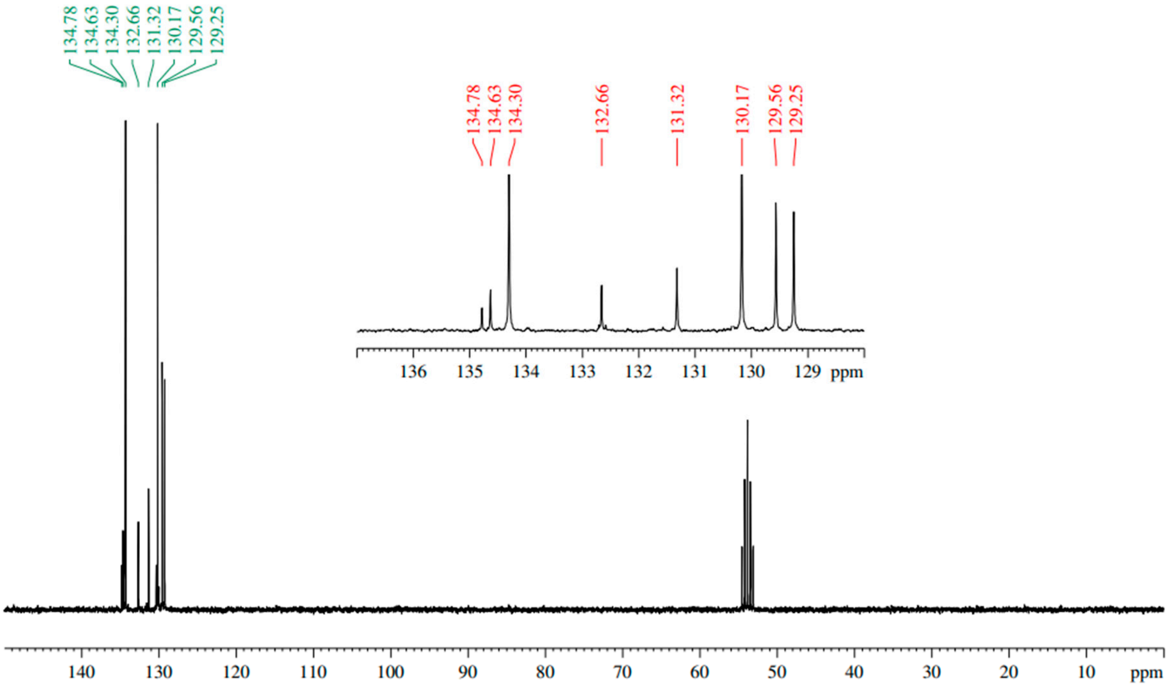

Figure S2: $^{13}C\{^{1}H\}$ NMR spectrum of compound **1**, CD_2Cl_2, 75 MHz, 25 °C.

2.2 Synthesis of Compound 2

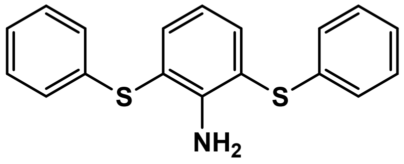

2

A solution of compound **1** (1.00 eq., 19.39 mmol, 6.00 g) in methanol (40 mL) was mixed with zinc powder (5.00 eq., 44.25 mmol, 2.93 g) and NH_4Cl (5.00 eq., 44.25 mmol, 2.40 g) and heated to reflux for 2 h. The mixture was filtered over celite, and the filtrate was concentrated. Ethyl acetate (200 mL) was added to the concentrated solution. The organic phase was extracted with a saturated aqueous solution of $NaHCO_3$ (100 mL) and dried over $MgSO_4$. The solvent was removed to obtain compound **2** in a yield of 92 % as an orange crystalline solid.

^{1}H NMR (300 MHz, CD_2Cl_2): δ (ppm) = 7.57 (d, ^3J = 7.76 Hz, 2H, aryl-CH), 7.26-7.07 (m, 10H, aryl-CH), 6.76 (t, ^3J=7.6 Hz, 1H, aryl-CH), 5.01 (br. s, 2H, NH_2).

^{13}C{^{1}H} NMR (75 MHz, CD_2Cl_2): δ (ppm) = 151.27 (aryl-C), 139.59 (aryl-C), 136.56 (aryl-C), 129.61 (aryl-C), 127.18 (aryl-C), 126.13 (aryl-C), 118.33 (aryl-C), 115.48 (aryl-C).

Elemental analysis: Calculated for $C_{18}H_{15}NS_2$: C, 69.87; H, 4.89; N, 4.53. Found: C, 70.15; H, 4.95; N, 4.65.

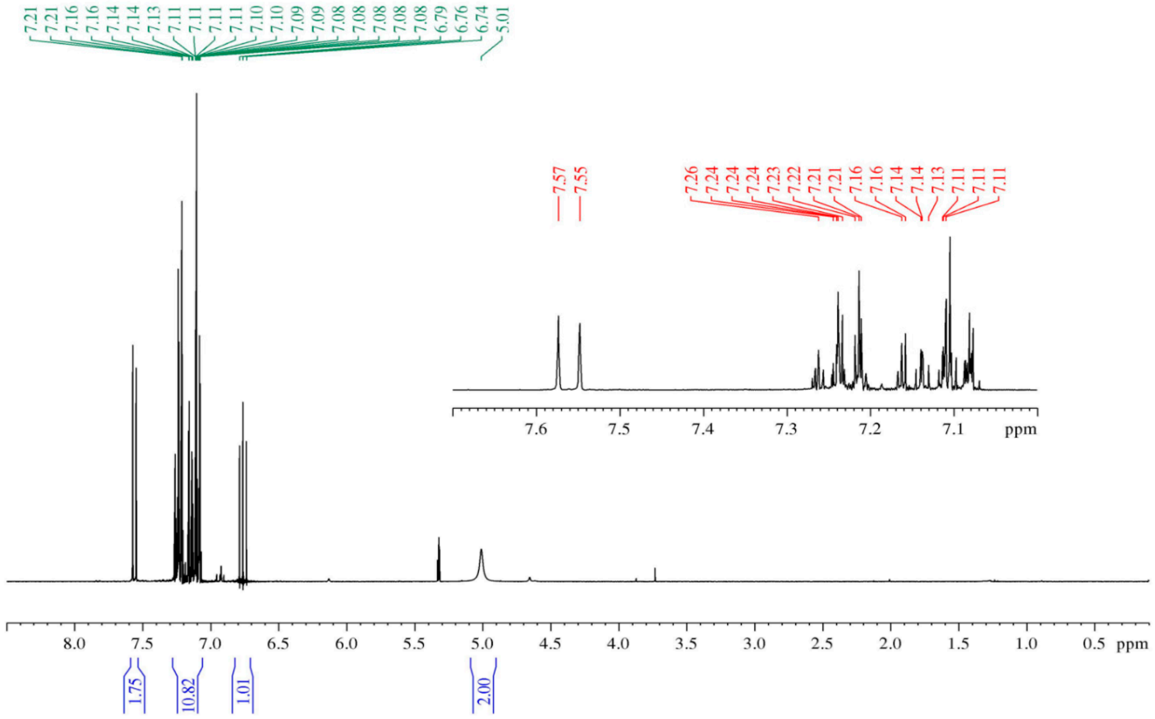

Figure S3: ^{1}H NMR spectrum of compound **2**, CD_2Cl_2, 300 MHz, 25 °C.

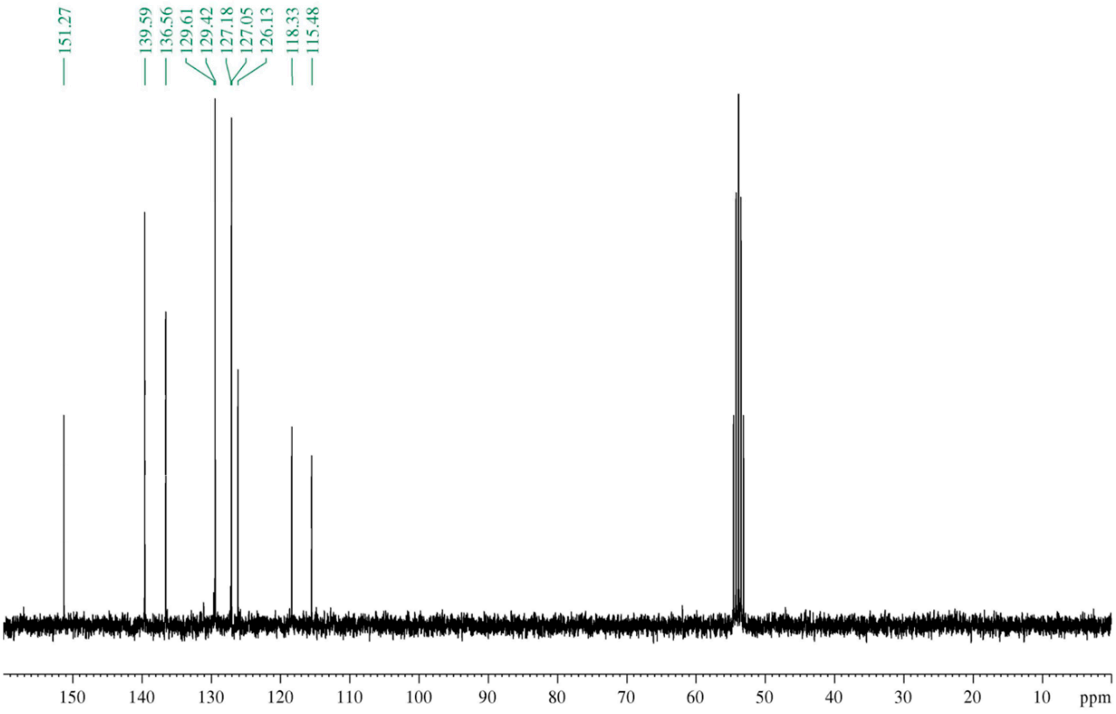

Figure S4: $^{13}C\{^1H\}$ NMR spectrum of compound **2**, CD_2Cl_2, 75 MHz, 25 °C.

2.3 Synthesis of Compound 3

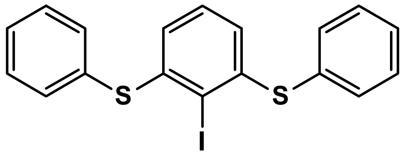

3

A mixture of compound **2** (1.00 eq., 3.23 mmol, 1.00 g) in acetonitrile (15-20 mL) and distilled water (5 mL) was added to a portion of concentrated HCl (aqueous, w = 37 %, 10 mL) at −10 °C. $NaNO_2$ (2.50 eq., 8.09 mmol, 0.56 g) in distilled water (10 mL) was added dropwise. Stirring was carried out for 1 h at −10 °C. KI (6.00 eq., 19.39 mmol, 3.22 g) was dissolved distilled water (50 mL) and added dropwise within 1 h. After stirring at room temperature overnight, the reaction was heated to 60 °C for 1 h. After cooling to ambient temperature dichloromethane (DCM, 250 mL) was added. The organic phase was washed with water (200 mL) and aqueous sodium thiosulfate solution ($Na_2S_2O_3$, w = 5 %, 100 mL). The organic phase was dried over $MgSO_4$ and the solvent was removed to afford compound **3** in a yield of 95 % as an orange powder.

^{1}H NMR (300 MHz, CD_2Cl_2): δ (ppm) = 7.45 (m, 10H, aryl-CH), 7.0 (m, 1H, aryl-CH), 6.64 (d, ^3J = 7.85 Hz, 2H, aryl-CH).

^{13}C{^{1}H} NMR (75 MHz, CD_2Cl_2): δ (ppm) = 145.09 (aryl-C), 134.19 (aryl-C), 134.09 (aryl-C), 130.16 (aryl-C), 129.18 (aryl-C), 129.13 (aryl-C), 126.11 (aryl-C). Due to the limited solubility of compound **7** in CD_2Cl_2, some of carbon signals cannot be detected.

Elemental analysis: Calculated for $C_{18}H_{13}IS_2$: C, 51.44; H, 3.12. Found: C, 51.05; H, 3.07.

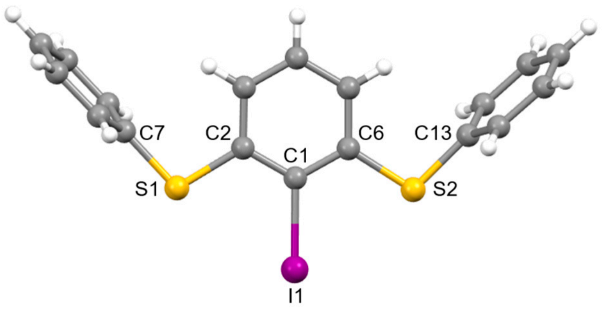

Figure S5: Molecular structure of compound **3**. Bond distances and bond angles are reported in Å or degree (°), respectively. S(1)-C(7) 1.7832(18), S(1)-C(2) 1.7790(18), C(1)-C(2) 1.402(2), I(1)-C(1) 2.1079(17), C(1)-C(6) 1.399(2), S(2)-C(6) 1.7787(18), S(2)-C(13) 1.7818(18), C(2)-S(1)-C(7) 102.53(8), C(6)-S(2)-C(13) 101.72(8), C(2)-C(1)-I(1) 119.33(12), C(6)-C(1)-I(1) 119.50(12).

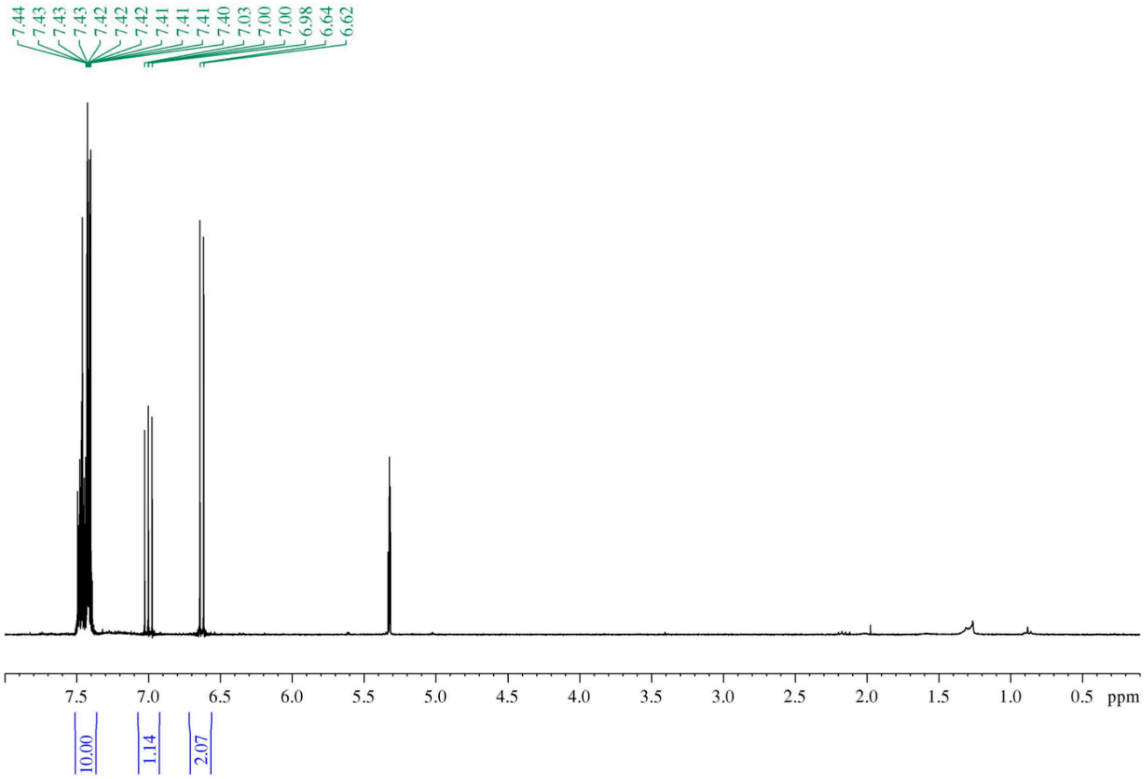

Figure S6: ^{1}H NMR spectrum of compound **3**, CD_2Cl_2, 300 MHz, 25 °C.

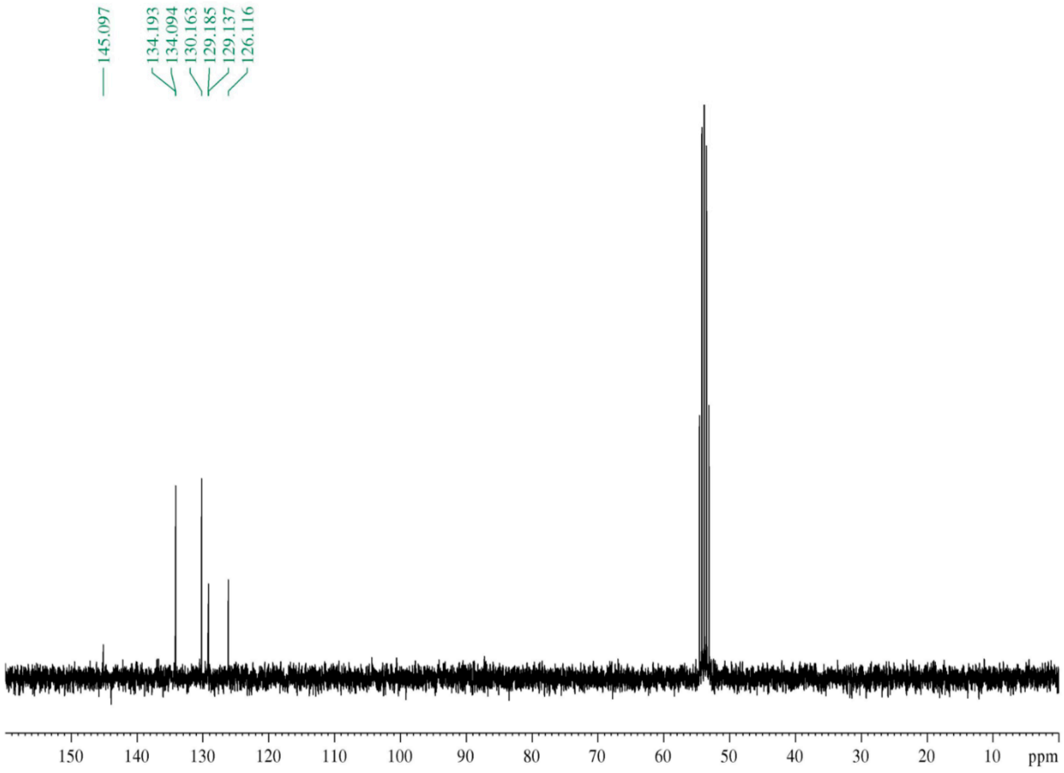

Figure S7: $^{13}C\{^{1}H\}$ NMR spectrum of compound **3**, CD_2Cl_2, 75 MHz, 25 °C.

2.4 Synthesis of Compound 4

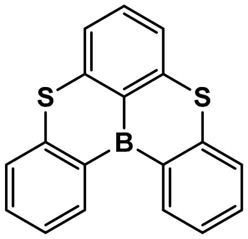

4

A solution of *n*-BuLi (1.05 eq., 2.5 M in hexanes, 1.70 mL, 4.2 mmol) was slowly added to a suspension of compound **3** (1.00 eq., 1.50 g, 4.0 mmol) in anhydrous *m*-xylene (50 mL) at −40 °C. The suspension was heated to 50 ºC and stirred for 1 h. The reaction was cooled to −30 ºC, at which boron tribromide (1.20 eq., 1.20 g, 0.50 mL, 4.8 mmol) was slowly added. The mixture was stirred at room temperature for 1 h. Hünig Base (diisopropylethyl amine, 2.00 eq., 1.10 g, 1.40 mL, 8.0 mmol) was then added at 0 °C. The reaction mixture was stirred at 125 ºC for 12 h. The suspension was cooled to room temperature. Sodium acetate aqueous solution (1 M, 50 mL) was added and the mixture was extracted with ethyl acetate (3 × 50 mL). The organic phase was dried over $MgSO_4$, and the solvent was removed under vacuum. The residue was crystallized from toluene by layering with *n*-pentane to obtain compound **4** in a yield of 81 % as a yellow crystalline material.

^{1}H NMR (300 MHz, $CDCl_3$): δ (ppm) = 8.30 (dd, ^3J = 7.84 Hz, 2H, aryl-CH), 7.70 (dd, ^3J = 7.93 Hz, 2H, aryl-CH), 7.50 (m, 7H, aryl-CH).

^{11}B{^{1}H} NMR (96 MHz, $CDCl_3$): δ (ppm) = 46.6 (s, $\omega_{1/2}$ = 683 Hz).

Elemental analysis: Calculated for $C_{18}H_{11}BS_2$: C, 71.54; H, 3.67; Found: C, 71.87; H, 3.72.

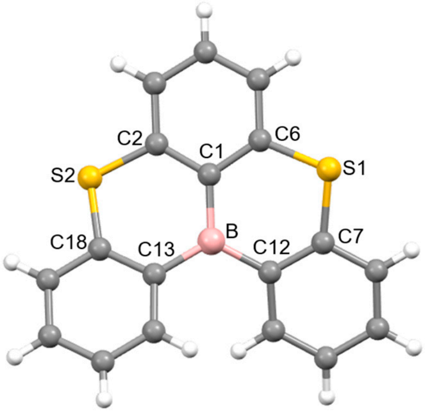

Figure S8: Molecular structure of compound **4**. Only the *P*-isomer is shown. Bond distances and bond angles are reported in Å or degree (°), respectively. S(1)-C(6) 1.7447(5), S(1)-C(7) 1.7465(5), S(2)-C(2) 1.7431(5), S(2)-C(18) 1.7465(5), C(1)-C(6) 1.4161(6), C(1)-C(2) 1.4160(7), C(1)-B(1) 1.5387(7), C(12)-B(1) 1.5518(7), C(13)-B(1) 1.5549(7), C(6)-S(1)-C(7) 105.62(2), C(2)-S(2)-C(18) 105.28(2),C(6)-C(1)-C(2) 115.88(4), C(6)-C(1)-B(1) 122.22(4), C(2)-C(1)-B(1) 121.85(4), C(1)-C(2)-S(2) 123.52(4), C(12)-C(7)-S(1) 124.05(4),C(7)-C(12)-B(1) 121.21(4), C(1)-B(1)-C(12) 118.74(4), C(1)-B(1)-C(13) 118.44(4), C(12)-B(1)-C(13) 122.81(4).

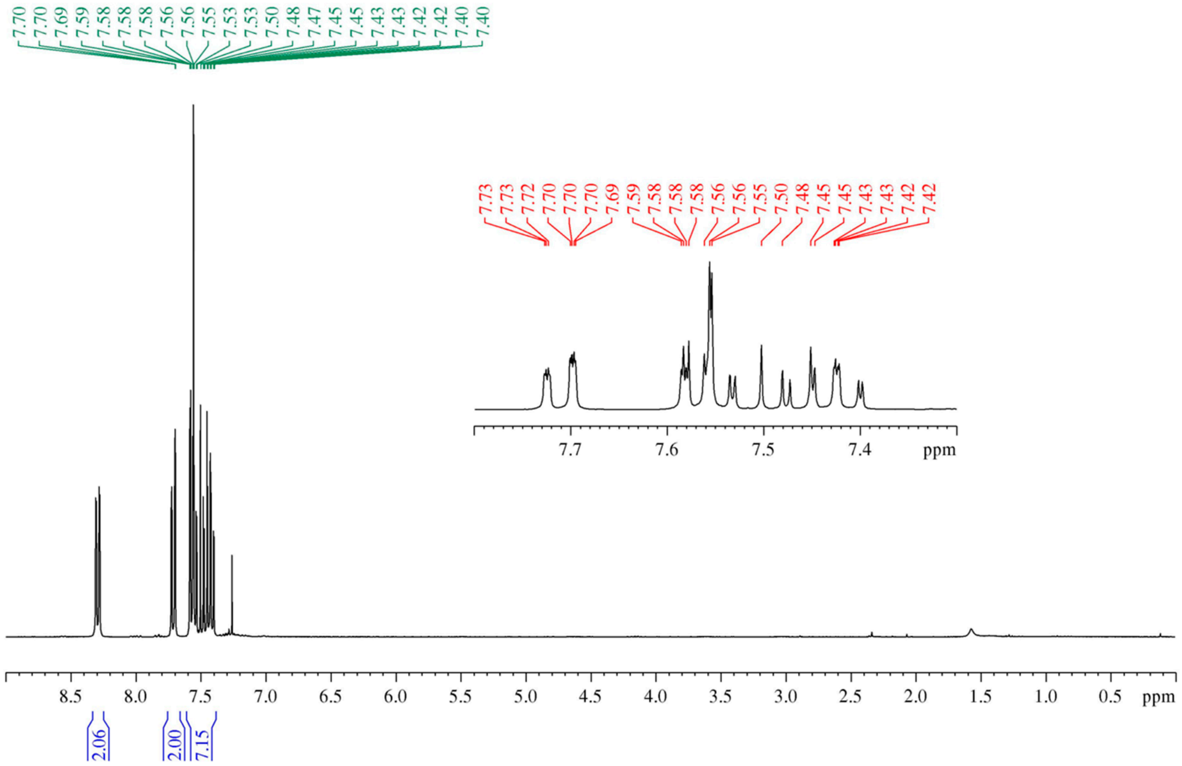

Figure S9: ^{1}H NMR spectrum of compound **4**, $CDCl_3$, 300 MHz, 25 °C.

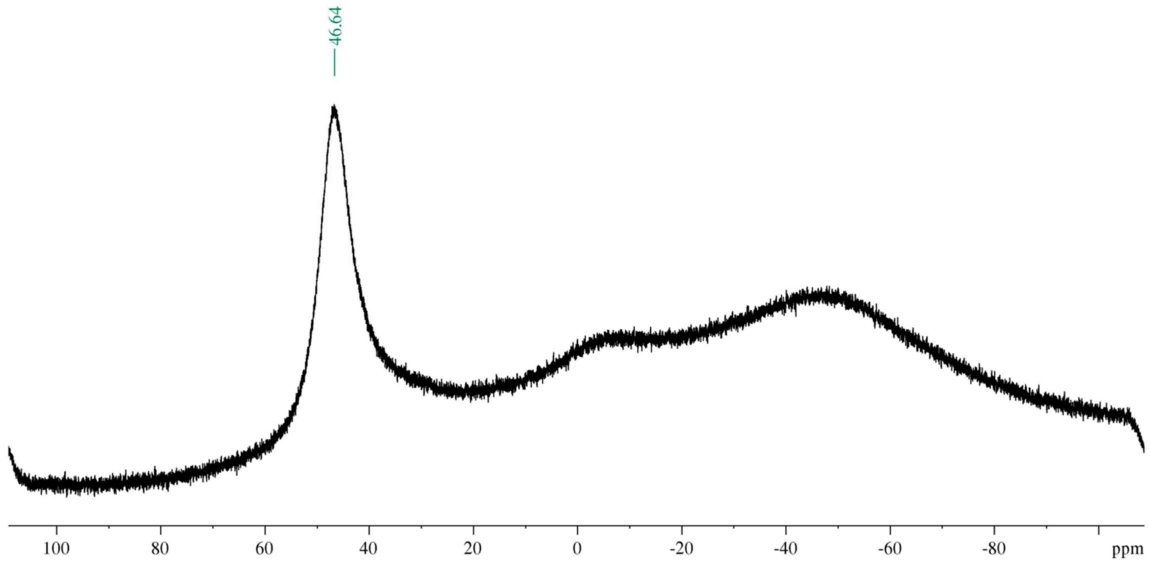

Figure S10: $^{11}B\{^{1}H\}$ NMR spectrum of compound **4**, $CDCl_3$, 96 MHz, 25 °C.

2.5 Synthesis of Compound 5

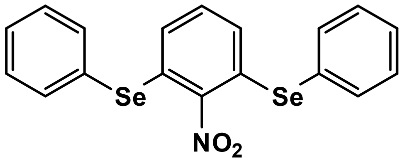

5

Sodium phenylselenide (NaSePh, 2.10 eq., 13.2 mmol, 2.36 g) and 1,3-difluoro-2-nitro-benzene (6.3 mmol, 1.00 g) were dissolved in anhydrous dimethylformamide (DMF, 5 mL). The reaction mixture was heated to 100 °C for 16 h. The mixture was cooled to ambient temperature, and dichloromethane (100 mL) and distilled water (100 mL) were added. The organic layer was dried over $MgSO_4$, and the solvent was removed to afford compound **5** in a yield of 91 % as a red powder.

^{1}H NMR (300 MHz, CD_2Cl_2): δ (ppm) = 7.70 (m, 4H, aryl-CH), 7.45 (m, 6H, aryl-CH), 6.90 (m, 1H, aryl-CH), 6.82 (dd, ^3J = 7.95 Hz, 2H, aryl-CH).

^{13}C{^{1}H} NMR (75 MHz, CD_2Cl_2): δ (ppm) = 137.64 (aryl-C), 137.39 (aryl-C), 136.94 (aryl-C), 132.21 (aryl-C), 130.43 (aryl-C), 130.15 (aryl-C), 129.61 (aryl-C), 128.56 (aryl-C).

Elemental analysis: Calculated for $C_{18}H_{13}NO_2Se_2$: C, 49.90; H, 3.02; N, 3.23; Found: C, 50.20; H, 2.95; N, 3.08.

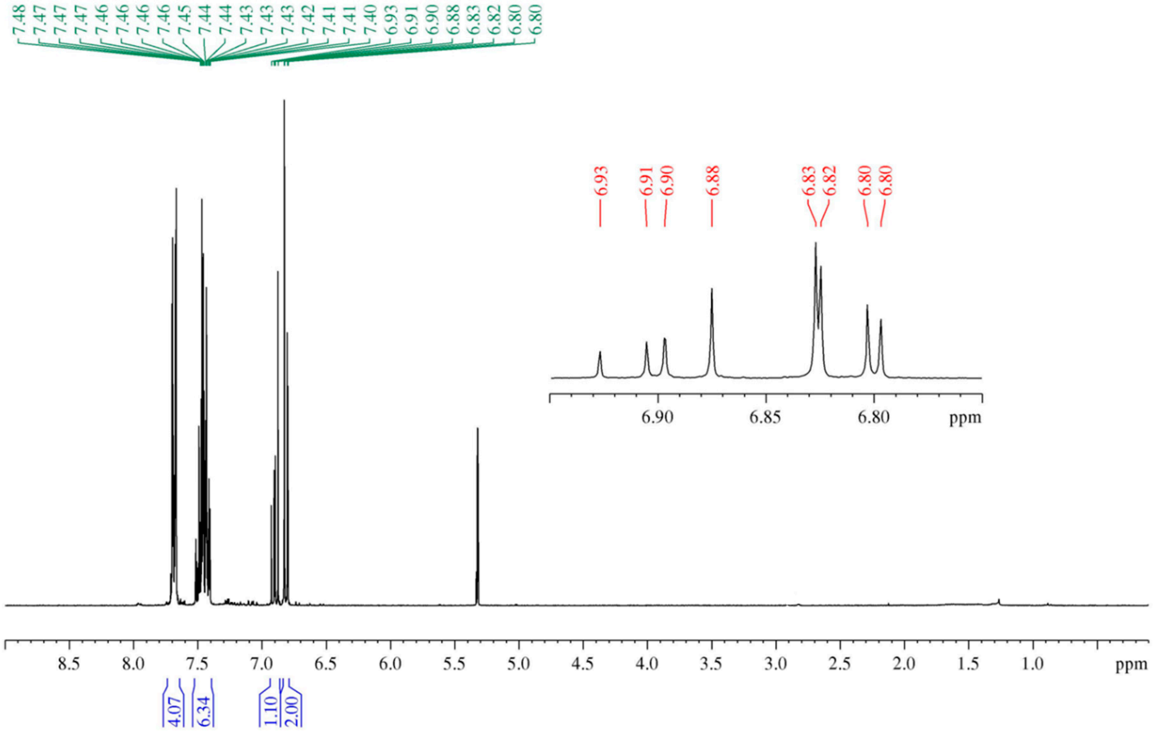

Figure S11: ^{1}H NMR spectrum of compound **5**, CD_2Cl_2, 300 MHz, 25 °C.

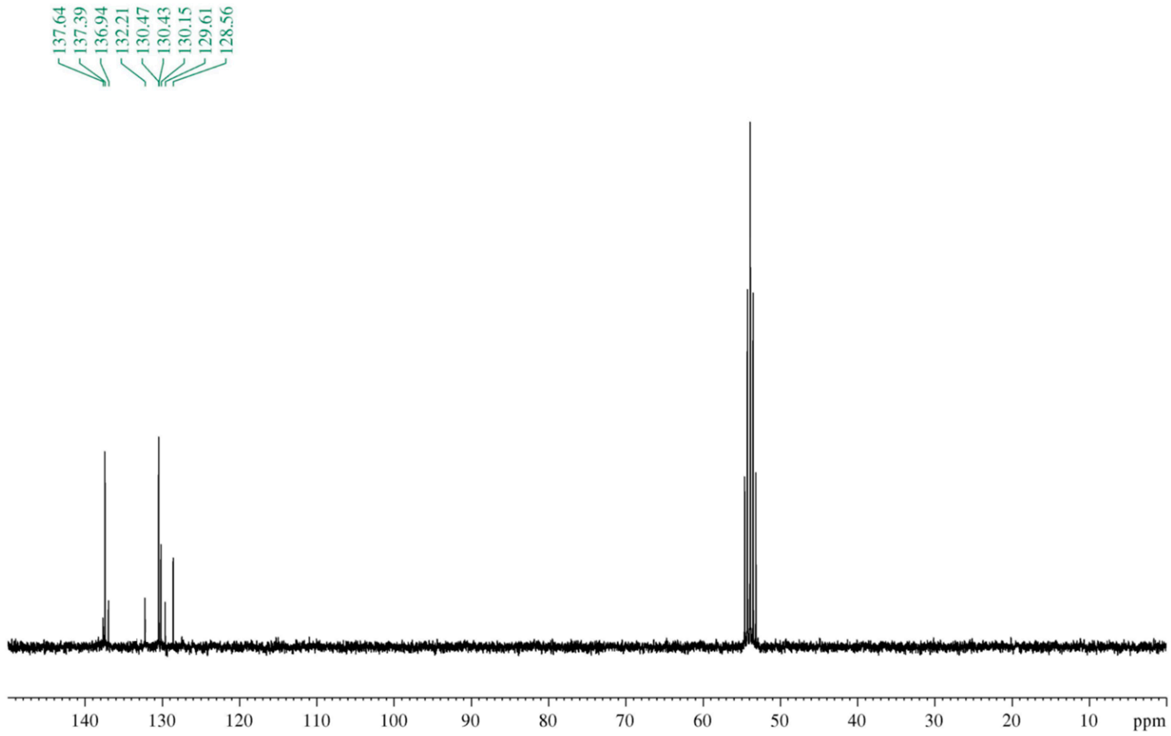

Figure S12: $^{13}C\{^{1}H\}$ NMR spectrum of compound **5**, CD_2Cl_2, 75 MHz, 25 °C.

2.6 Synthesis of Compound 6

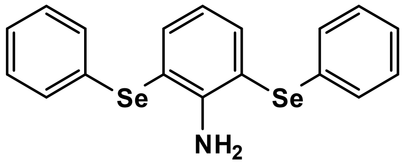

6

A solution of compound **5** (1.00 eq., 5.8 mmol, 2.50 g) in methanol (40 mL) was mixed with zinc powder (5.00 eq., 28.9 mmol, 1.88 g) and NH_4Cl (5.00 eq., 28.9 mmol, 1.54 g) and heated to reflux for 0.5 h. The mixture was filtered over celite, and the filtrate was concentrated. Ethyl acetate (200 mL) was added to the concentrated solution. The organic phase was extracted with a saturated aqueous solution of $NaHCO_3$ (100 mL) and dried over $MgSO_4$. The solvent was removed to obtain compound **6** in a yield of 90 % as a brownish crystalline solid.

^{1}H NMR (300 MHz, CD_2Cl_2): δ (ppm) = 7.66 (d, ^3J = 7.84 Hz, 2H, aryl-CH), 7.22 (m, 10H, aryl-CH), 6.66 (m, 1H, aryl-CH), 5.03 (br. s, 2 H, NH_2).

^{13}C{^{1}H} NMR (75 MHz, CD_2Cl_2): δ (ppm) = 150.69 (aryl-C), 140.64 (aryl-C), 131.69 (aryl-C), 129.96 (aryl-C), 129.67 (aryl-C), 126.87 (aryl-C), 118.92 (aryl-C), 113.23 (aryl-C).

Elemental analysis: Calculated for $C_{18}H_{15}NSe_2$: C, 53.61; H, 3.75; N, 3.47; Found: C, 54.01; H, 3.89; N, 3.59.

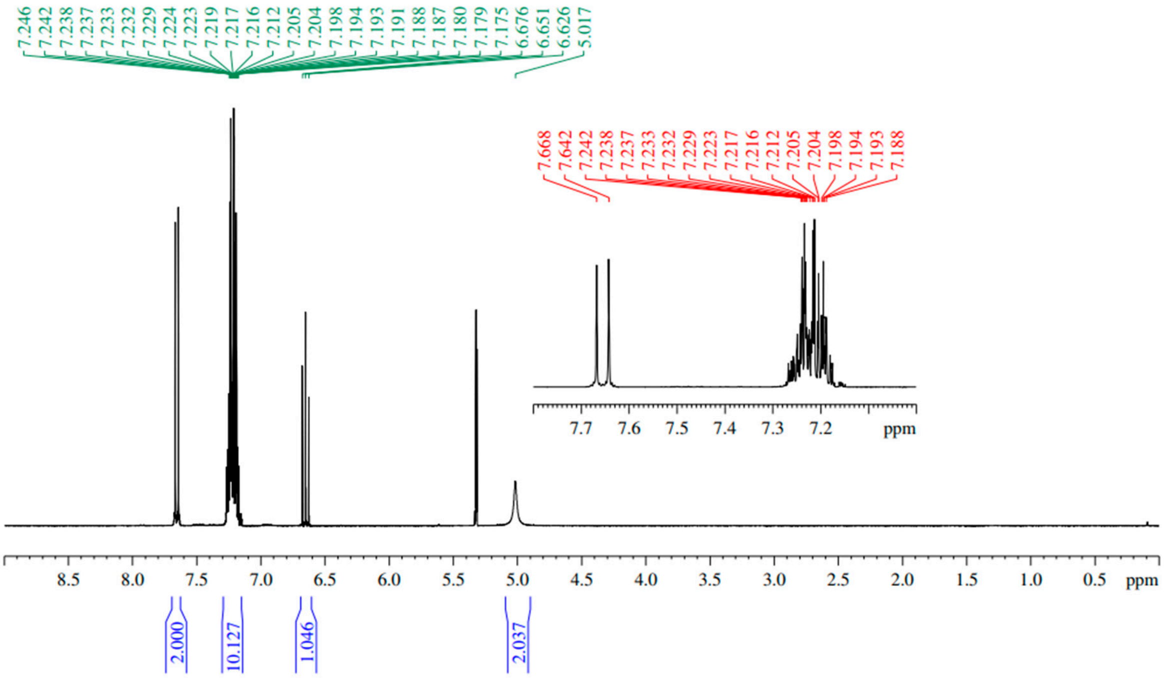

Figure S13: ^{1}H NMR spectrum of compound **6**, CD_2Cl_2, 300 MHz, 25 °C.

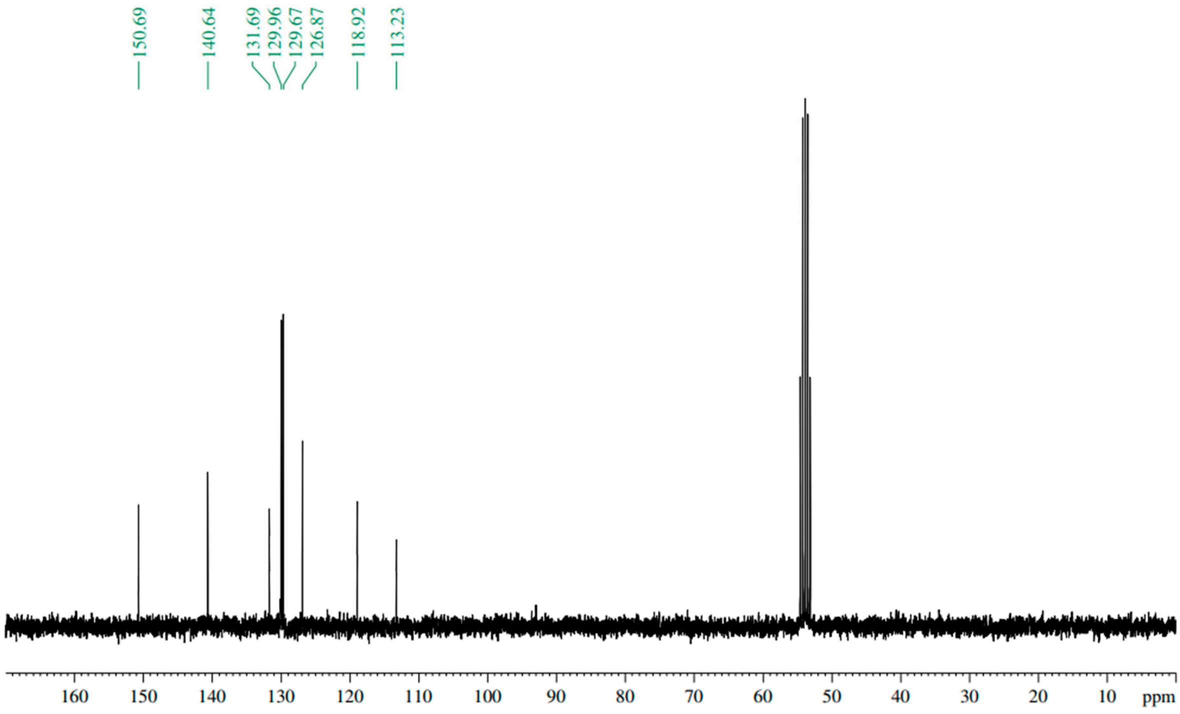

Figure S14: $^{13}C\{^1H\}$ NMR spectrum of compound **6**, CD_2Cl_2, 75 MHz, 25 °C.

2.7 Synthesis of Compound 7

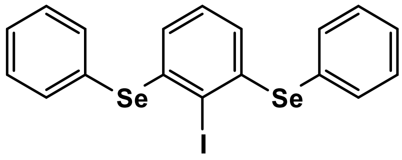

7

A mixture of compound **6** (1.00 eq., 5.0 mmol, 2.00 g) in acetonitrile (15-20 mL) and distilled water (5 mL) was added to a portion of concentrated HCl (aqueous, w = 37 %, 10 mL) at −10 °C. $NaNO_2$ (4.00 eq., 18.8 mmol, 1.36 g) in distilled water (10 mL) was added dropwise. Stirring was carried out for 1 h at −10 °C. KI (6.00 eq., 29.9 mmol, 4.94 g) was dissolved distilled water (50 mL) and added dropwise within 1 h. After stirring at room temperature overnight, the reaction was heated to 60 °C for 1 h. After cooling to ambient temperature dichloromethane (DCM, 250 mL) was added. The organic phase was washed with water (200 mL) and aqueous sodium thiosulfate solution ($Na_2S_2O_3$, w = 5 %, 100 mL). The organic phase was dried over $MgSO_4$ and the solvent was removed to afford compound **7** in a yield of 88 % as an orange powder.

^{1}H NMR (300 MHz, CD_2Cl_2): δ (ppm) = 7.66 (dd, ^3J = 7.48 Hz, 4H, aryl-CH), 7.42 (m, 6H, aryl-CH), 6.87 (t, ^3J = 8.14 Hz, 1H, aryl-CH), 6.61 (dd, ^3J = 7.82 Hz, 2H, aryl-CH).

^{13}C{^{1}H} NMR (75 MHz, CD_2Cl_2): δ (ppm) = 136.62 (aryl-C), 130.51 (aryl-C), 129.66 (aryl-C), 127.60 (aryl-C). Due to the limited solubility of compound **7** in CD_2Cl_2, some of carbon signals cannot be detected.

Elemental analysis: Calculated for $C_{18}H_{13}ISe_2$: C, 42.05; H, 2.55; I, 24.68; Found: C, 42.13; H, 2.83.

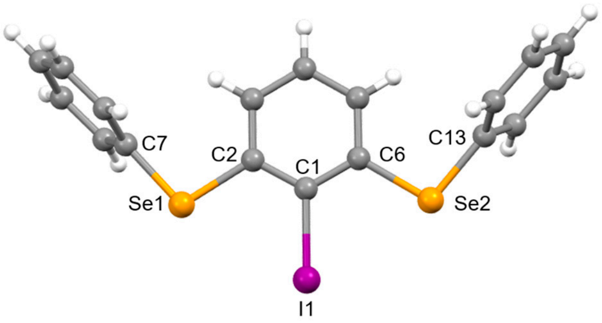

Figure S15: Molecular structure of compound **7**. Bond distances and bond angles are reported in Å or degree (°), respectively. I(1)-C(1) 2.107(5), Se(1)-C(7) 1.919(5), Se(1)-C(2) 1.929(5), Se(2)-C(6) 1.923(5), Se(2)-C(13) 1.925(5), C(1)-C(6) 1.393(7), C(1)-C(2) 1.393(7), C(7)-Se(1)-C(2) 99.7(2), C(6)-Se(2)-C(13) 99.3(2), C(6)-C(1)-C(2) 121.1(5), C(6)-C(1)-I(1) 119.5(4), C(2)-C(1)-I(1) 119.3(4).

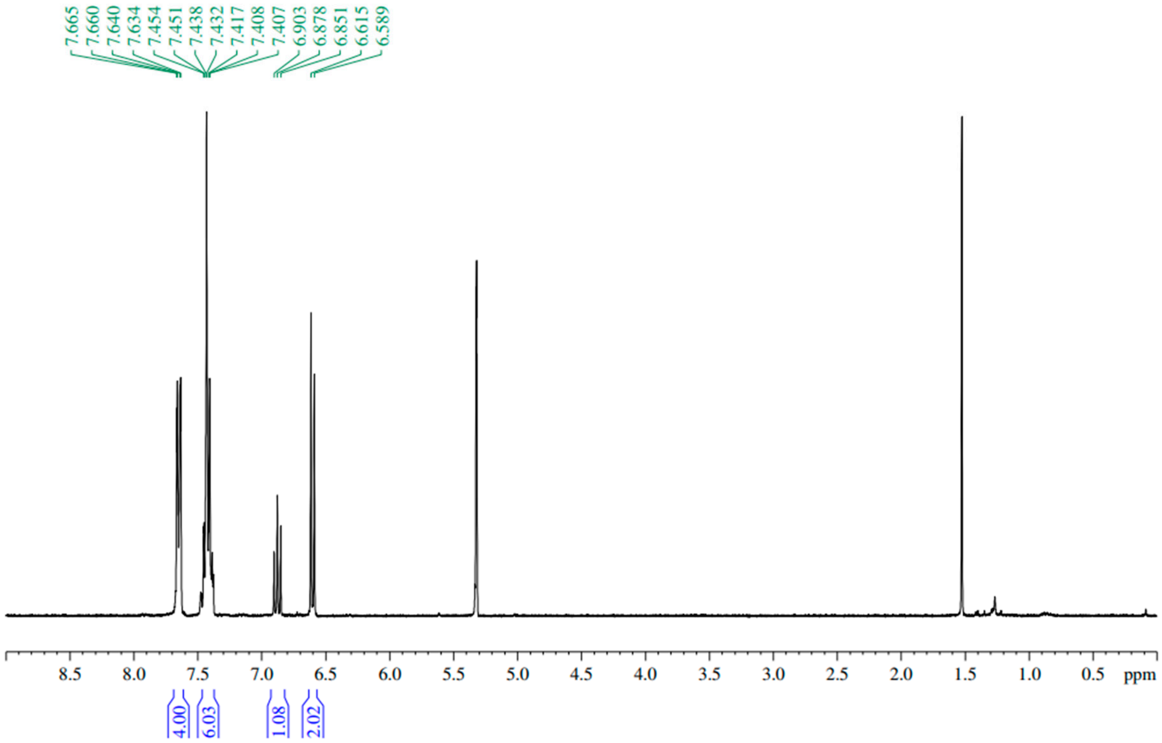

Figure S16: ^{1}H NMR spectrum of compound **7**, CD_2Cl_2, 300 MHz, 25 °C.

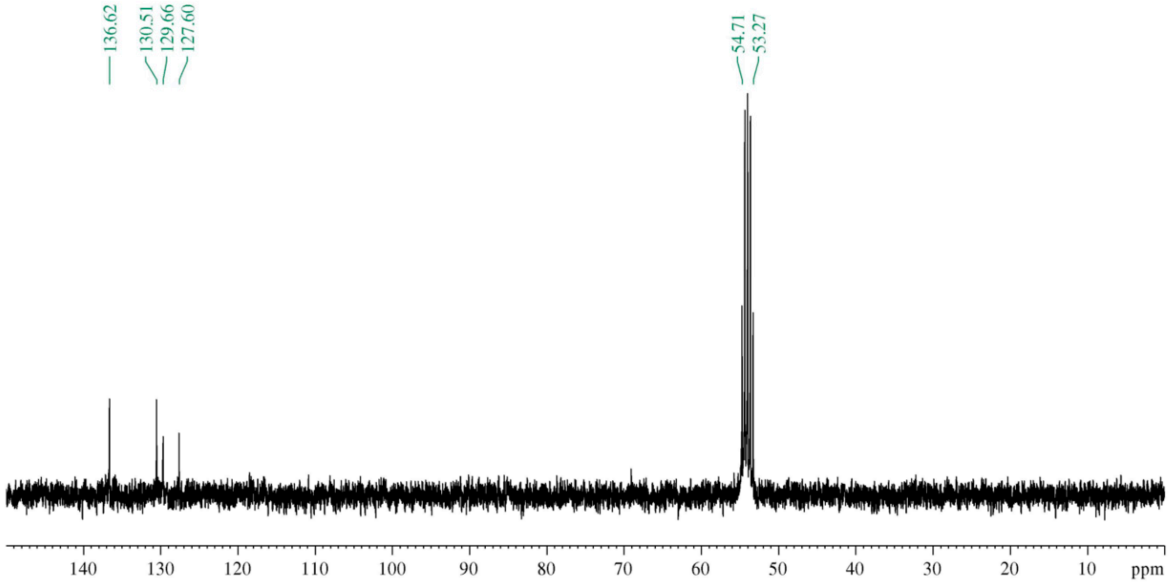

Figure S17: $^{13}C\{^{1}H\}$ NMR spectrum of compound **7**, CD_2Cl_2, 75 MHz, 25 °C.

2.8 Synthesis of Compound 8

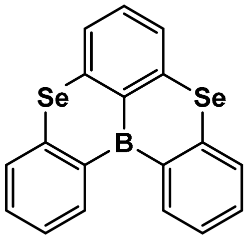

8

A solution of *n*-BuLi (1.05 eq., 2.5 M in hexanes, 1.70 mL, 4.2 mmol) was slowly added to a suspension of compound **7** (1.00 eq., 1.50 g, 4.0 mmol) in anhydrous *m*-xylene (50 mL) at −30 °C. The suspension was heated to 50 ºC and stirred for 1 h. The reaction was cooled to −30 ºC, at which boron tribromide (1.20 eq., 1.20 g, 0.50 mL, 4.8 mmol) was slowly added. The mixture was stirred at room temperature for 1 h. Hünig Base (diisopropylethyl amine, 2.00 eq., 1.10 g, 1.40 mL, 8.0 mmol) was then added at 0 °C. The reaction mixture was stirred at 125 ºC for 12 h. The suspension was cooled to room temperature. Sodium acetate aqueous solution (1 M, 50 mL) was added and the mixture was extracted three times with ethyl acetate (3 × 50 mL). The organic phase was dried over $MgSO_4$, and the solvent was removed under vacuum. The residue was crystallized from toluene by layering with *n*-pentane to obtain compound **8** in a yield of 67 % as a yellow crystalline material.

^{1}H NMR (300 MHz, CD_2Cl_2): δ (ppm) = 8.02 (d, ^{3}J = 7.7 Hz, 2H, aryl-CH), 7.81 (d, ^{3}J = 7.7 Hz, 2H, aryl-CH), 7.72 (d, 2H, ^{3}J = 7.7 Hz, aryl-CH), 7.51 (dt, 2H, ^{3}J = 7.64 Hz, ^{5}J = 1.4 Hz, aryl-CH), 7.41 (t, 2H, ^{3}J = 7.43 Hz, aryl-CH), 7.34 (t, 1H, ^{3}J = 7.82 Hz, aryl-CH).

^{11}B{^{1}H} NMR (96 MHz, CD_2Cl_2): δ (ppm) = 52.3 (s, $\omega_{½}$ = 785 Hz).

^{13}C{^{1}H} NMR (75 MHz, CD_2Cl_2): δ (ppm) = 143.05 (aryl-C), 142.50 (aryl-C), 140.81 (aryl-CH), 131.24 (aryl-CH), 130.22 (aryl-CH), 127.96 (aryl-CH), 125.86 (aryl-CH), 125.39 (aryl-CH). Not observed B-*C*.

^{77}Se{^{1}H} NMR (95 MHz, CD_2Cl_2): δ (ppm) = 413.2

Elemental analysis: Calculated for $C_{18}H_{13}BSe_2$: C, 54.32; H, 3.29; Found. C, 54.19; H, 3.26.

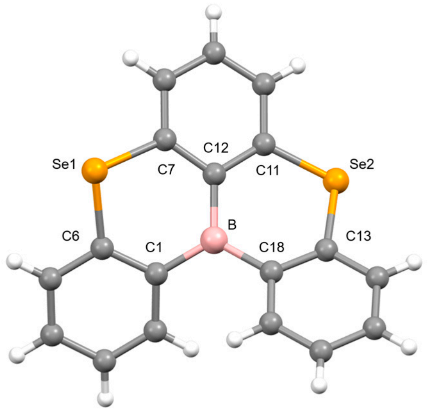

Figure S18: Molecular structure of compound **8**. Only the *P*-isomer is shown. Bond distances and bond angles are reported in Å or degree (°), respectively. Se(1)-C(6) 1.903(3), Se(1)-C(7) 1.906(3), Se(2)-C(13) 1.901(3), Se(2)-C(11) 1.901(3), C(1)-C(6) 1.408(4), C(1)-B(1) 1.557(4), C(12)-B(1) 1.540(5), C(18)-B(1) 1.560(5), C(6)-Se(1)-C(7) 99.36(14), C(13)-Se(2)-C(11) 100.39(13), C(6)-C(1)-B(1) 122.2(3), C(11)-C(12)-B(1) 122.1(3), C(7)-C(12)-B(1) 122.6(3), C(18)-C(13)-Se(2) 122.3(2), C(13)-C(18)-B(1) 123.1(3), C(12)-B(1)-C(1) 119.1(3), C(12)-B(1)-C(18) 119.6(3), C(1)-B(1)-C(18) 121.3(3).

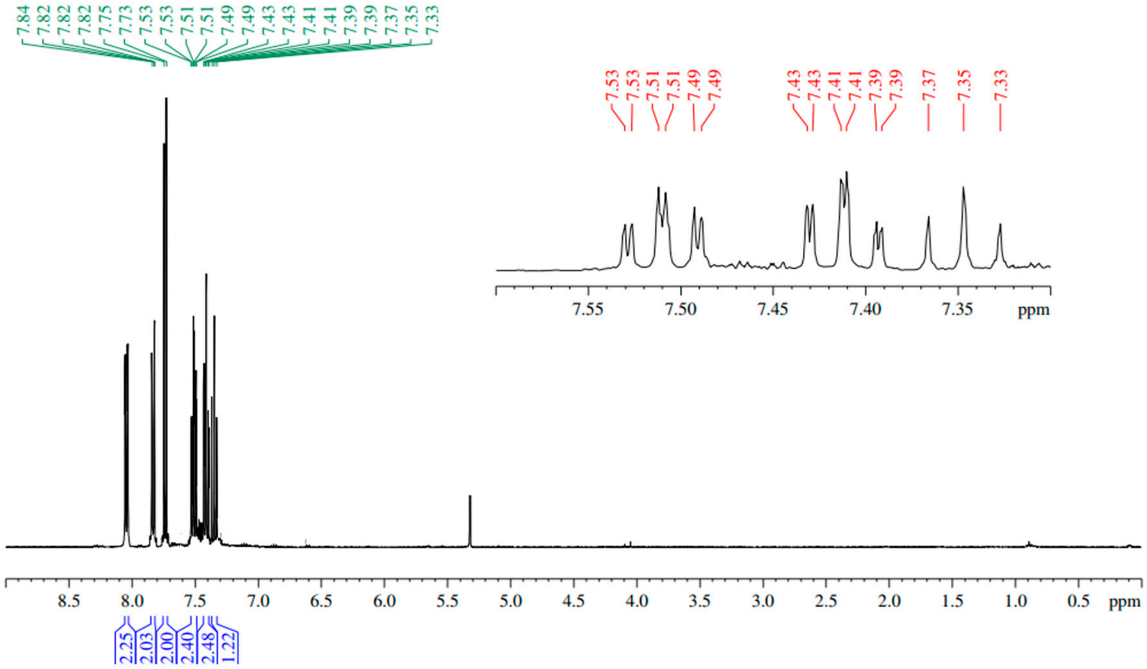

Figure S19: ^{1}H NMR spectrum of compound **8**, CD_2Cl_2, 300 MHz, 25 °C.

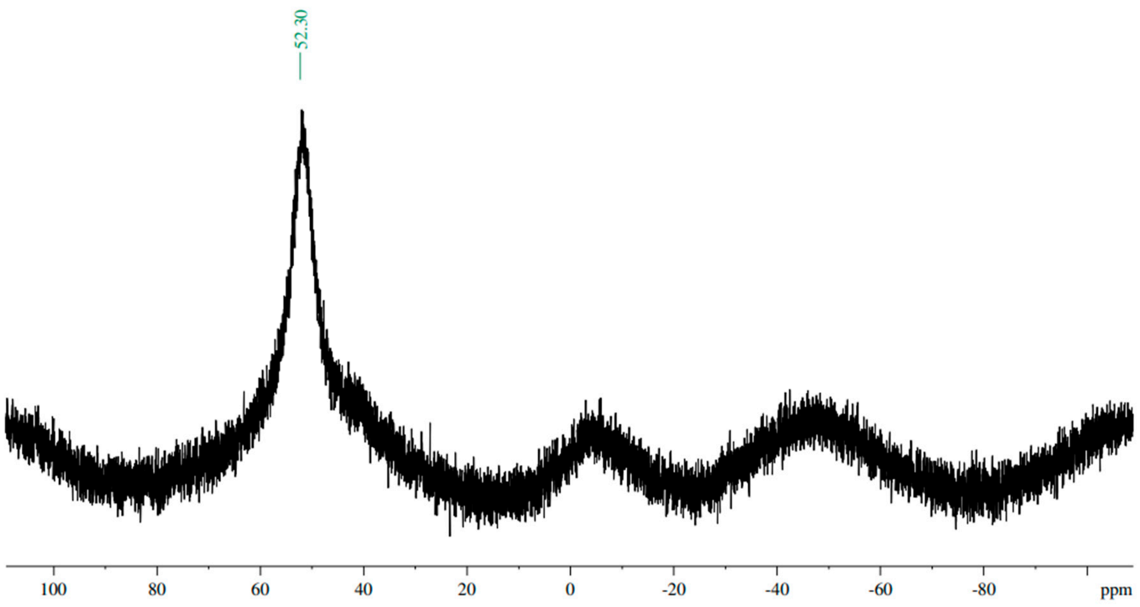

Figure S20: $^{11}B\{^{1}H\}$ NMR spectrum of compound **8**, CD_2Cl_2, 96 MHz, 25 °C.

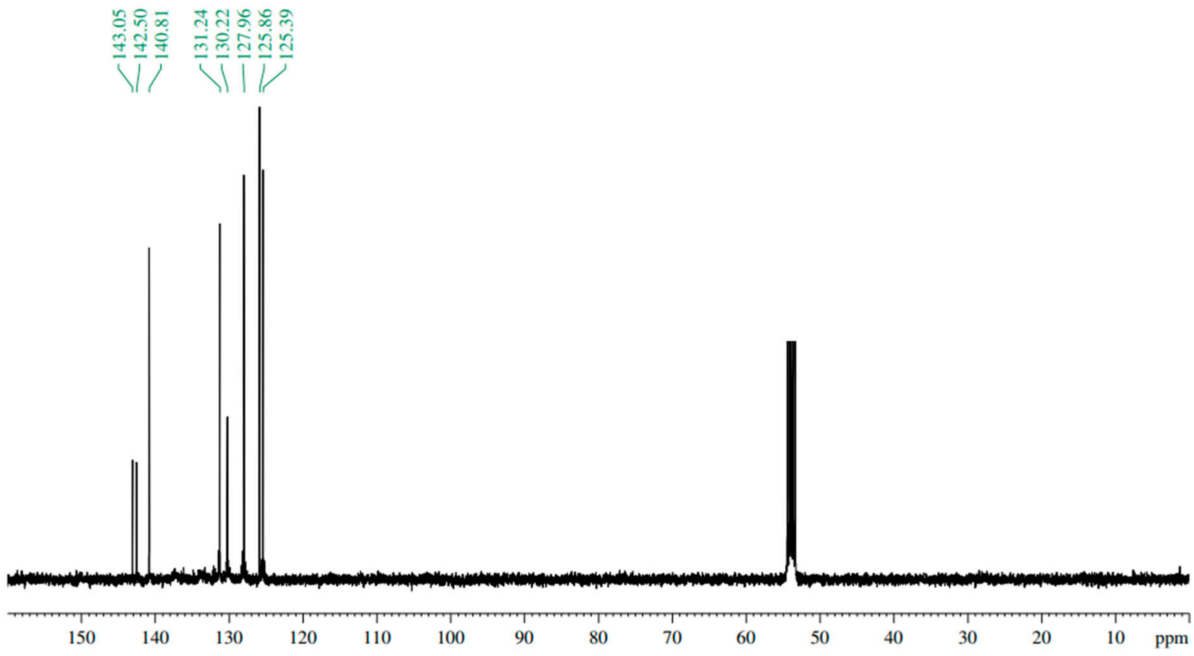

Figure S21: $^{13}C\{^{1}H\}$ NMR spectrum of compound **8**, CD_2Cl_2, 75 MHz, 25 °C.

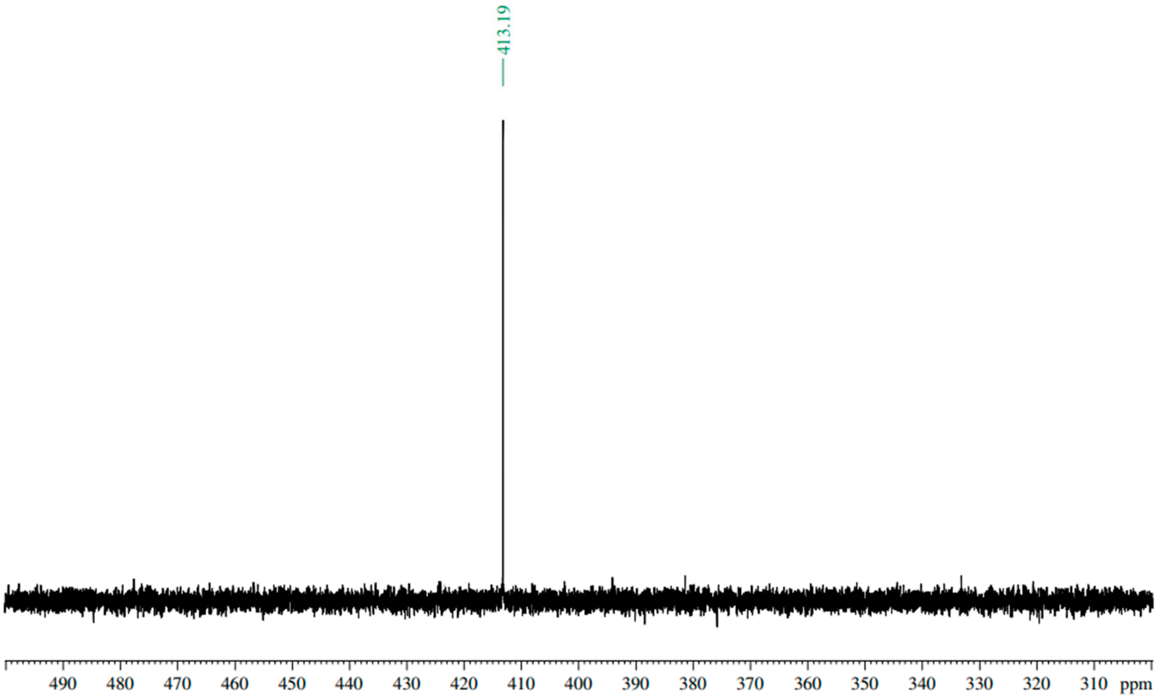

Figure S22: $^{77}Se\{^{1}H\}$ NMR spectrum of compound **8**, CD_2Cl_2, 95 MHz, 25 °C.

2.9 Synthesis of Compound 9

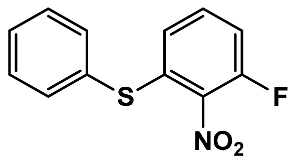

9

Thiophenol (0.50 eq., 2.57 mL, 25.1 mmol) and 1,3-difluoro-2-nitrobenzene (1.00 eq., 2.00 g, 12.6 mmol) were added to a stirred suspension of K_2CO_3 (3.00 eq., 5.20 g, 37.7 mmol) in anhydrous dimethylformamide (DMF, 20 mL) at 0 °C. After stirring for 16 h at 25 °C, dichloromethane (DCM, 100 mL) was added. The organic phases were washed with water and a saturated aqueous solution of $NaHCO_3$ (100 mL each). The organic layer was dried over $MgSO_4$ and filtered. Solvent evaporation afforded compound **9** in a yield of 86 % (calculated based on thiophenol) as a yellow crystalline material.

^{1}H NMR (300 MHz, CD_2Cl_2): δ (ppm) = 7.51 (m, 5H, aryl-CH), 7.31 (ddd, 1H, aryl-CH), 7.06 (m, 1H, aryl-CH), 6.85 (m, 1H, aryl-CH).

^{13}C{^{1}H} NMR (75 MHz, CD_2Cl_2): δ (ppm) = 155.23 ($^1J_{C,F}$ = 259 Hz, aryl-C-F), 136.38 (aryl-C), 134.80 (aryl-C), 132.65 ($^3J_{C,F}$ = 8 Hz, aryl-CH), 131.59 (aryl-C), 130.34 (aryl-C), 129.99 (aryl-C) 126.24 (aryl-C), 114.72 ($^2J_{C,F}$ = 20 Hz, aryl-CH). Not observed *C*-NO_2

Elemental analysis: Calculated for $C_{12}H_8FNO_2S$: C, 57.82; H, 3.24; N, 5.62; Found: C, 58.03; H, 3.10; N, 5.50.

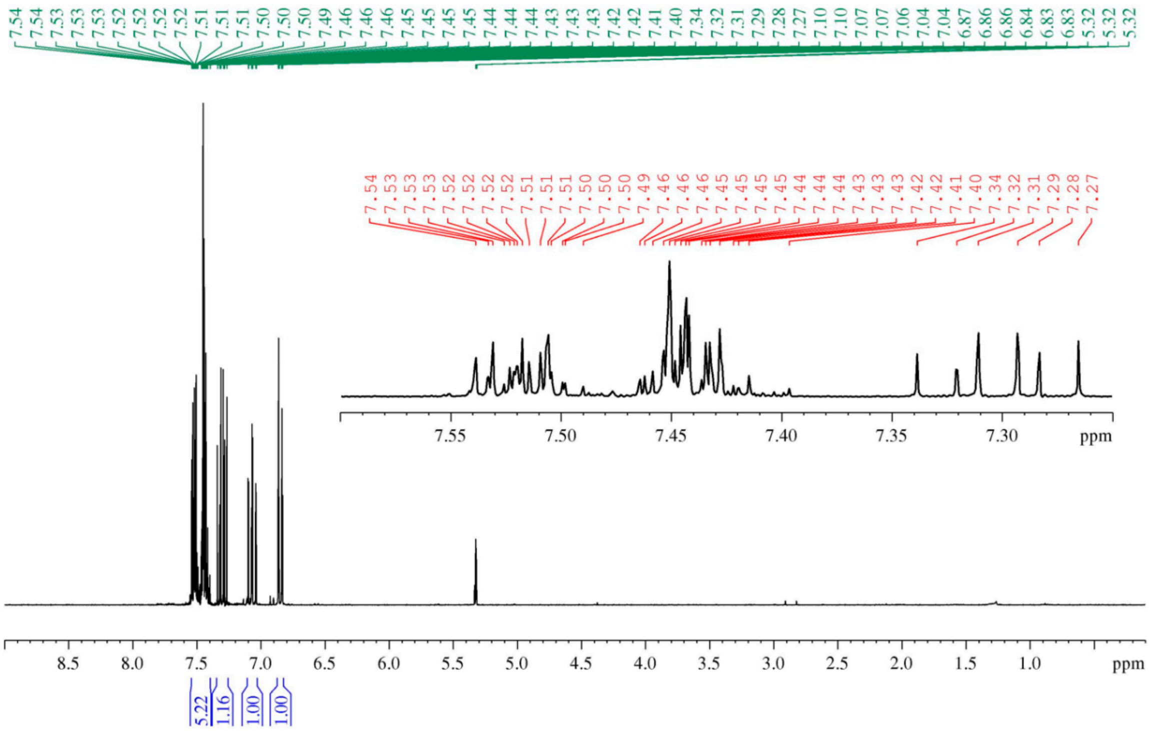

Figure S23: ^{1}H NMR spectrum of compound **9**, CD_2Cl_2, 300 MHz, 25 °C.

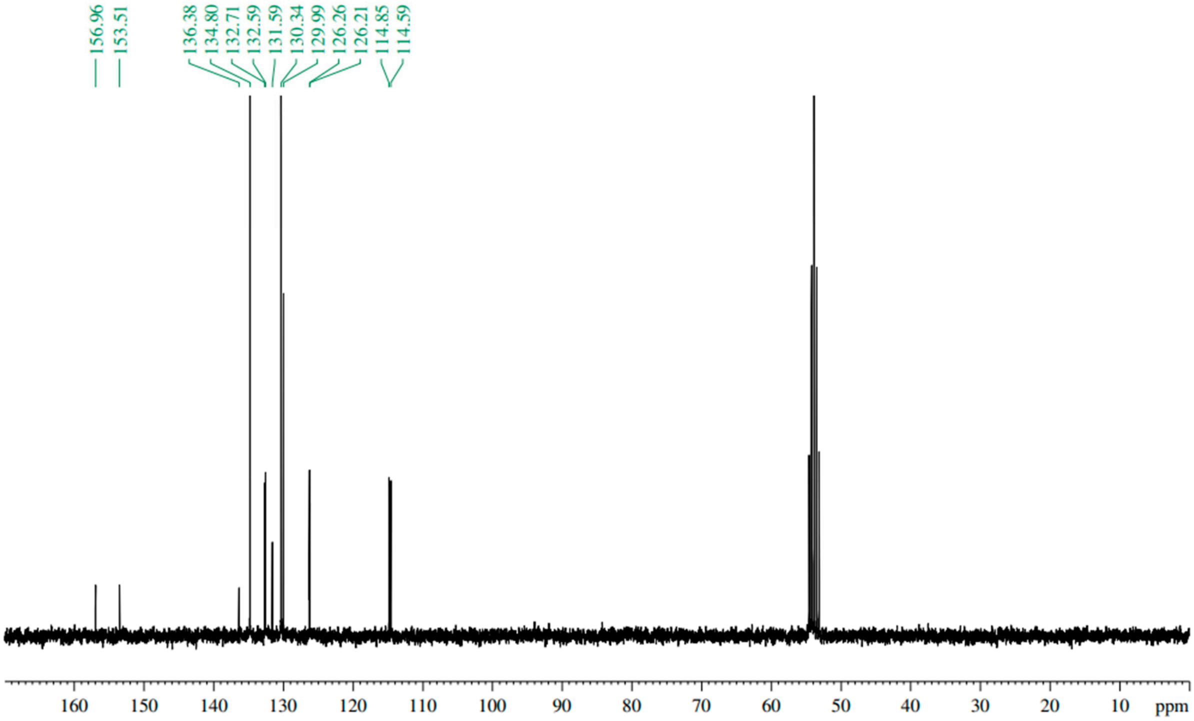

Figure S24: $^{13}C\{^{1}H\}$ NMR spectrum of compound **9**, CD_2Cl_2, 75 MHz, 25 °C.

2.10. Synthesis of Compound 10

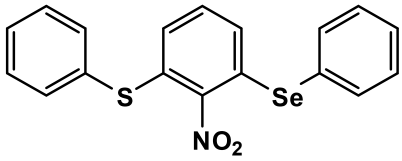

10

Compound **9** (1.00 eq., 4.0 mmol, 1.00 g) and sodium phenylselenide (NaSePh, 1.00 eq., 4.0 mmol, 0.72 g) were dissolved in anhydrous dimethylformamide (DMF, 15 mL). The reaction mixture was heated to 100 °C 16 h. The mixture was cooled to ambient temperature, and dichloromethane (100 mL) and distilled water (100 mL) were added. The organic layer was dried over $MgSO_4$, and the solvent was removed to afford compound **10** in a yield of 88 % as an orange crystalline solid.

^{1}H NMR (300 MHz, CD_2Cl_2): δ (ppm) = 7.65 (m, 2H, aryl-CH), 7.44 (m, 8H, aryl-CH), 6.98 (m, 1H, aryl-CH), 6.84 (dd, ^3J = 8.02 Hz, 1H, aryl-CH), 6.78 (dd, ^3J = 7.93 Hz, 1H, aryl-CH).

^{13}C{^{1}H} NMR (75 MHz, CD_2Cl_2): δ (ppm) = 136.84 (aryl-C), 135.23 (aryl-C), 131.77 (aryl-C), 130.33 (aryl-C), 129.93 (aryl-C), 129.16 (aryl-C), 128.03 (aryl-C). Due to the limited solubility of compound 10 in CD2Cl2, some of carbon signals cannot be detected

Elemental analysis: Calculated for $C_{18}H_{13}NO_2SSe$: C, 55.96; H, 3.39; N, 3.63; Found: C, 56.17; H, 3.38; N, 3.54.

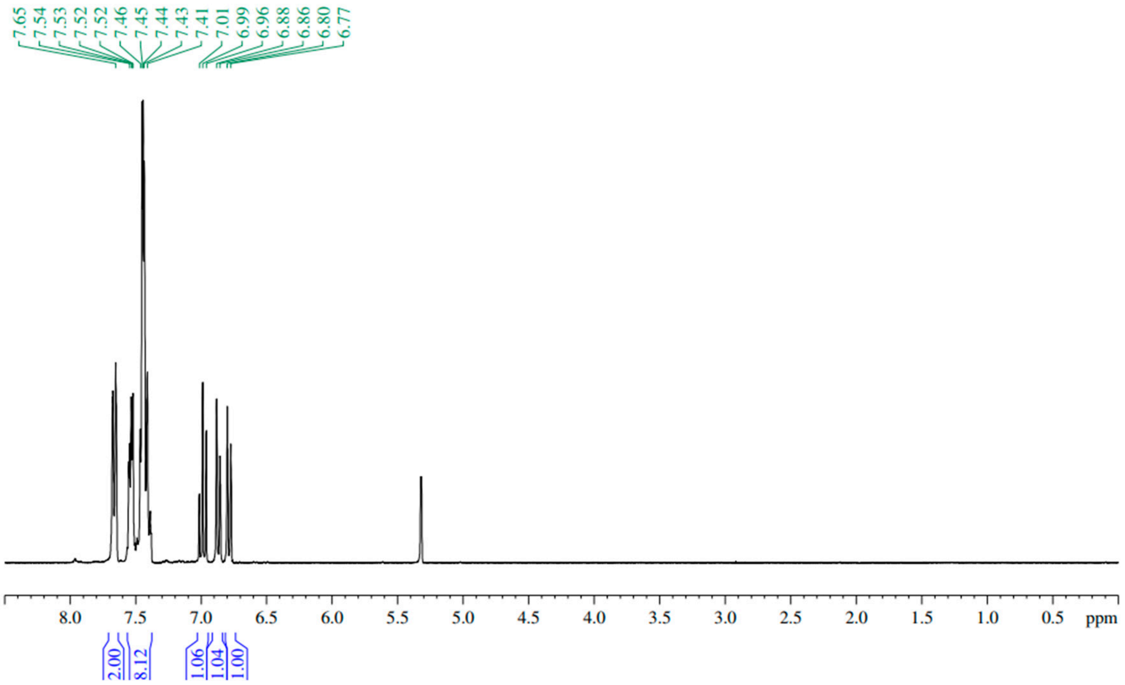

Figure S25: ^{1}H NMR spectrum of compound **10**, CD_2Cl_2, 300 MHz, 25 °C.

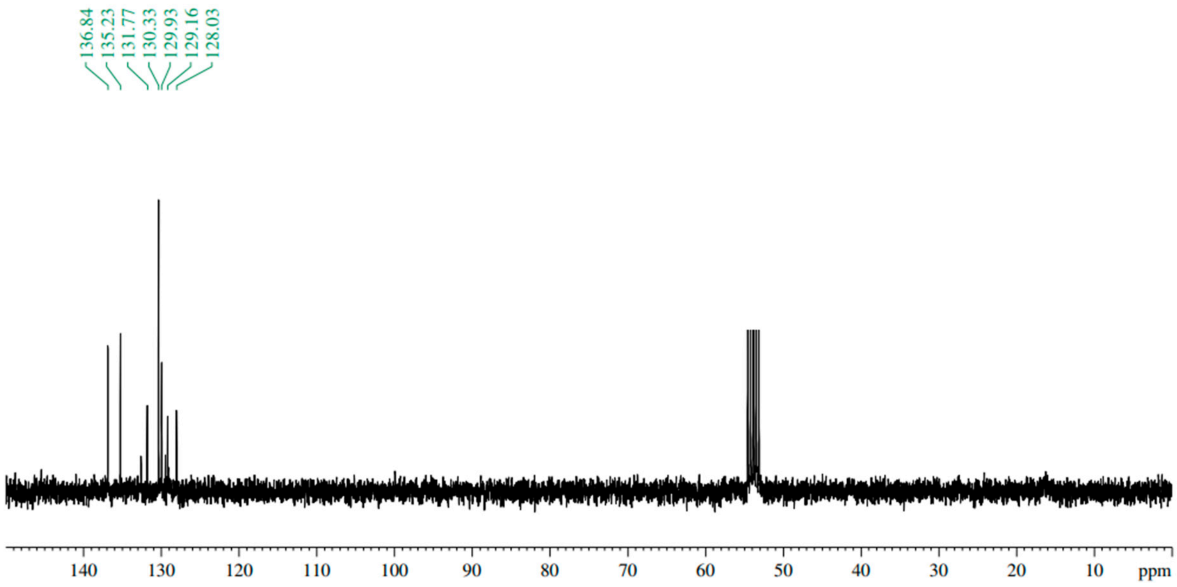

Figure S26: $^{13}C\{^{1}H\}$ NMR spectrum of compound **10**, CD_2Cl_2, 75 MHz, 25 °C.

2.11. Synthesis of Compound 11

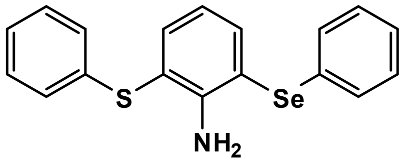

11

A solution of compound **10** (1.00 eq., 2.6 mmol, 1.00 g) in methanol (20 mL) was mixed with zinc powder (5.00 eq., 12.9 mmol, 0.86 g) and NH_4Cl (5.00 eq., 12.5 mmol, 0.70 g) and heated to reflux for 1 h. The mixture was filtered over celite, and the filtrate was concentrated. Ethyl acetate (50 mL) was added to the concentrated solution. The organic phase was extracted with a saturated aqueous solution of $NaHCO_3$ (50 mL) and dried over $MgSO_4$. The solvent was removed to obtain compound **11** in a yield of 91 % as an orange crystalline solid.

^{1}H NMR (300 MHz, CD_2Cl_2): δ (ppm) = 7.70 (dd, ^3J= 7.38 Hz, 1H, aryl-CH), 7.59 (dd, ^3J=7.70 Hz, 1H, aryl-CH), 7.23 (m, 10H, aryl-CH), 6.74 (m, 1H, aryl-CH), 5.02 (br. s, 2H, NH_2).

^{13}C{^{1}H} NMR (75 MHz, CD_2Cl_2): δ (ppm) = 151.01 (aryl-C), 140.70 (aryl-C), 139.45 (aryl-C), 136.67 (aryl-C), 131.64 (aryl-C), 130.03 (aryl-C), 129.68 (aryl-C), 129.42 (aryl-C), 127.13 (aryl-C), 126.90 (aryl-C), 126.13 (aryl-C), 118.64 (aryl-C). 115.95 (aryl-C), 115.03 (aryl-C).

Elemental analysis: Calculated for: $C_{18}H_{15}NSSe$: C, 60.67; H, 4.24; N, 3.93; Found: C, 60.98; H, 4.13; N, 4.17.

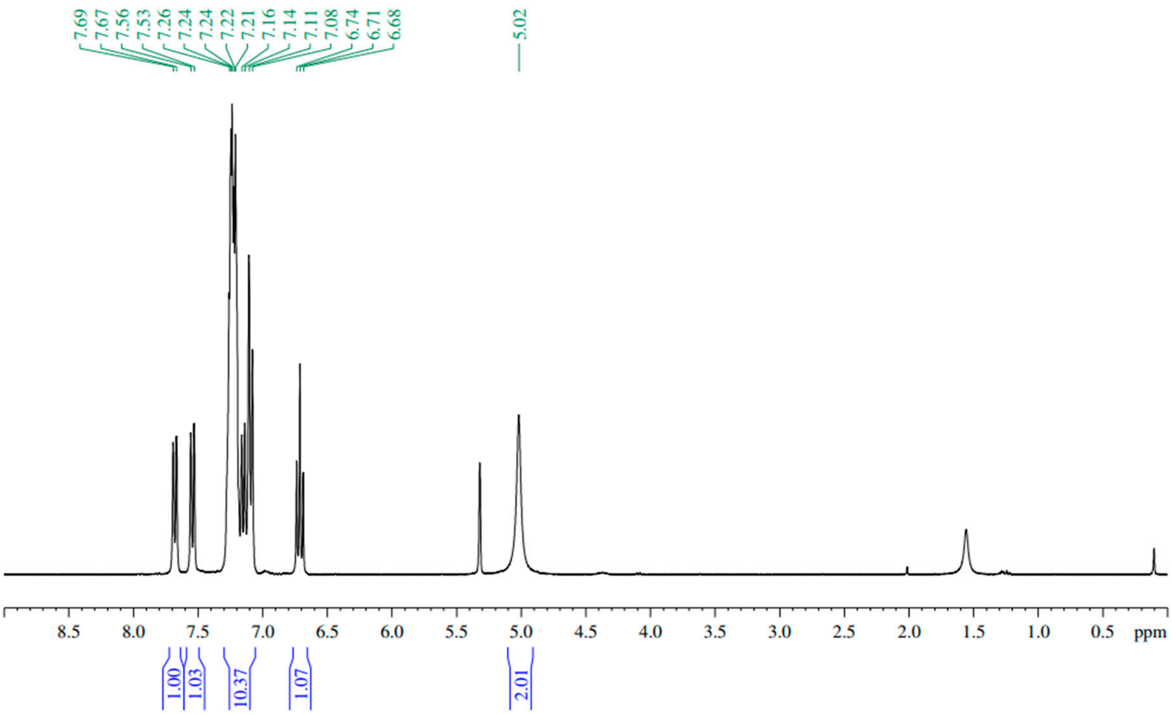

Figure S27: ^{1}H NMR spectrum of compound **11**, CD_2Cl_2, 300 MHz, 25 °C.

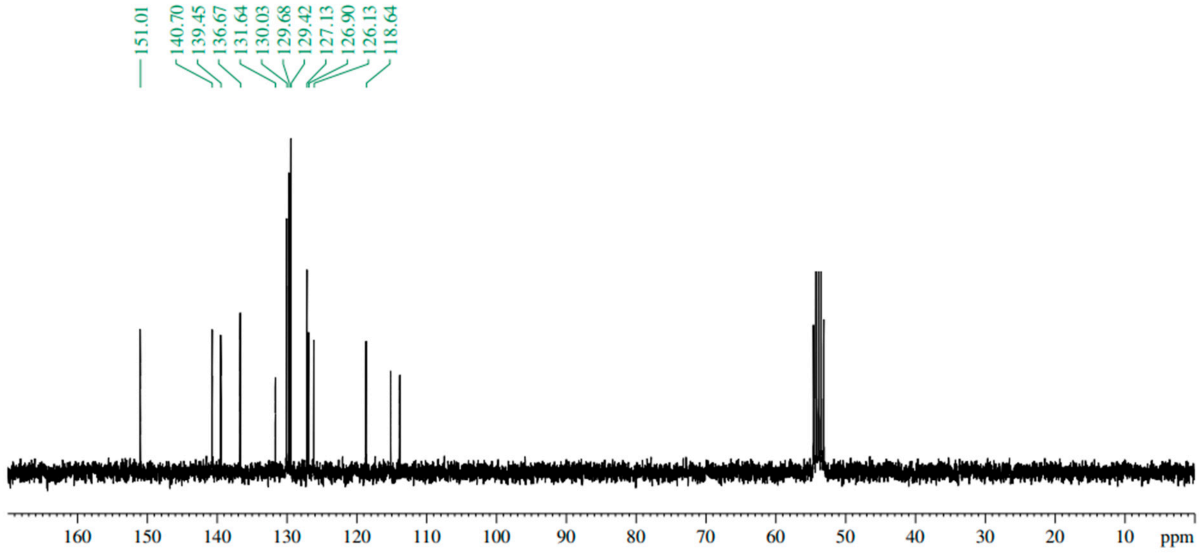

Figure S28: $^{13}C\{^{1}H\}$ NMR spectrum of compound **11**, CD_2Cl_2, 75 MHz, 25 °C.

2.12. Synthesis of Compound 12

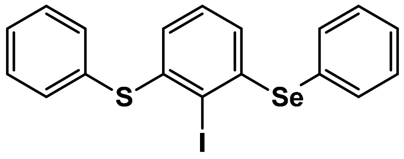

12

A mixture of compound **11** (1.00 eq., 2.8 mmol, 1.00 g) in acetonitrile (15-20 mL) and distilled water (5 mL) was added to a portion of concentrated HCl (aqueous, w = 37 %, 10 mL) at −10 °C. $NaNO_2$ (4.00 eq., 11.2 mmol, 0.77 g) in distilled water (10 mL) was added dropwise. Stirring was carried out for 1 h at −10 °C. KI (6.00 eq., 16.86 mmol, 2.80 g) was dissolved distilled water (50 mL) and added dropwise within 1 h. After stirring at room temperature overnight, the reaction was heated to 60 °C for 1 h. After cooling to ambient temperature dichloromethane (DCM, 150 mL) was added. The organic phase was washed with water (100 mL) and aqueous sodium thiosulfate solution ($Na_2S_2O_3$, w = 5 %, 100 mL). The organic phase was dried over $MgSO_4$ and the solvent was removed to afford compound **12** in a yield of 92 % as an orange powder. Compound **12** shows limited solubility in CD_2Cl_2, which impeded the measurement of more concentrated NMR spectra.

^{1}H NMR (300 MHz, CD_2Cl_2): δ (ppm) = 7.67 (m, 2H, aryl-CH), 7.41 (m, 8H, aryl-CH), 6.94 (tr., ^3J = 20.08 Hz, 1H, aryl-CH), 6.67 (dd, ^3J = 7.92 Hz, 1H, aryl-CH), 6.56 (dd, ^3J = 7.64 Hz, 1H, aryl-CH).

^{13}C{^{1}H} NMR (75 MHz, CD_2Cl_2): δ (ppm) = 144.61 (aryl-C), 143.68 (aryl-C), 136.66 (aryl-C), 134.37 (aryl-C), 134.09 (aryl-C), 133.80 (aryl-C), 131.05 (aryl-C), 130.38 (aryl-C), 130.12 (aryl-C), 129.59 (aryl-C), 129.34 (aryl-C), 128.99 (aryl-C), 127.19 (aryl-C), 126.43 (aryl-C).

Elemental analysis. Calculated for: $C_{18}H_{13}ISSe$: C, 46.27; H, 2.80; Found: C, 46.18; H, 2.86.

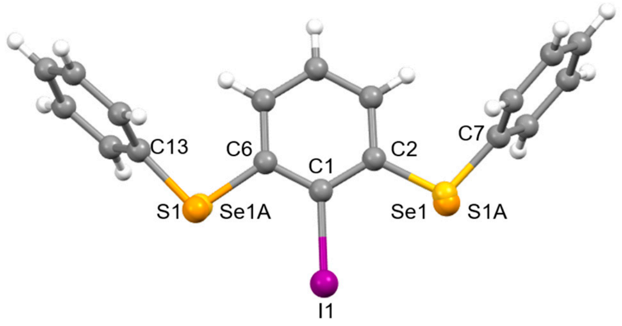

Figure S29: Molecular structure of compound **12**. The positions of sulfur (S1 and S1A) and selenium (Se1 and Se1A) were refined at fixed occupancy of 0.5 each. Bond distances and bond angles are reported in Å or degree (°), respectively. I(1)-C(1) 2.1052(15), C(1)-C(2) 1.401(2), C(1)-C(6) 1.404(2), C(2)-S(1A) 1.774(10), C(2)-Se(1) 1.910(4), C(6)-S(1) 1.779(9), C(6)-Se(1A) 1.907(4), C(7)-S(1A) 1.868(11), C(7)-Se(1) 1.870(4), C(13)-S(1) 1.777(9), C(13)-Se(1A) 1.896(4), C(2)-C(1)-C(6) 121.08(14), C(2)-C(1)-I(1) 119.34(11), C(6)-C(1)-I(1) 119.57(11), C(1)-C(2)-S(1A) 118.4(4), C(1)-C(2)-Se(1) 117.75(17).

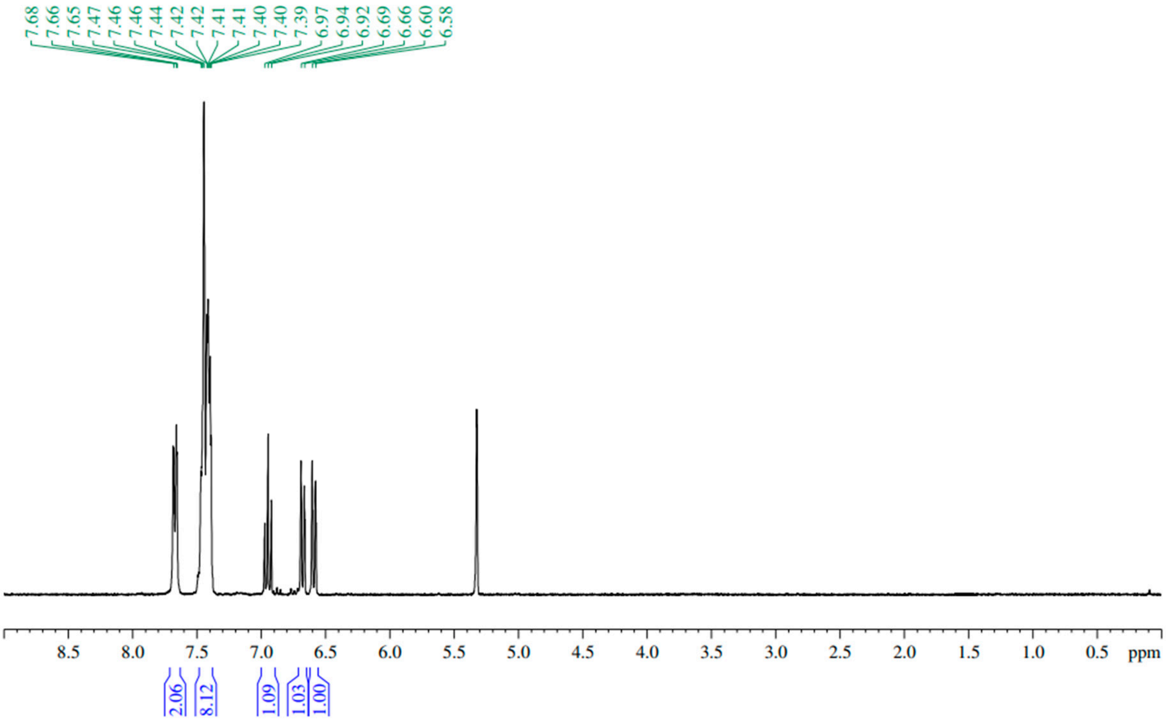

Figure S30: ^{1}H NMR spectrum of compound **12**, CD_2Cl_2, 300 MHz, 25 °C.

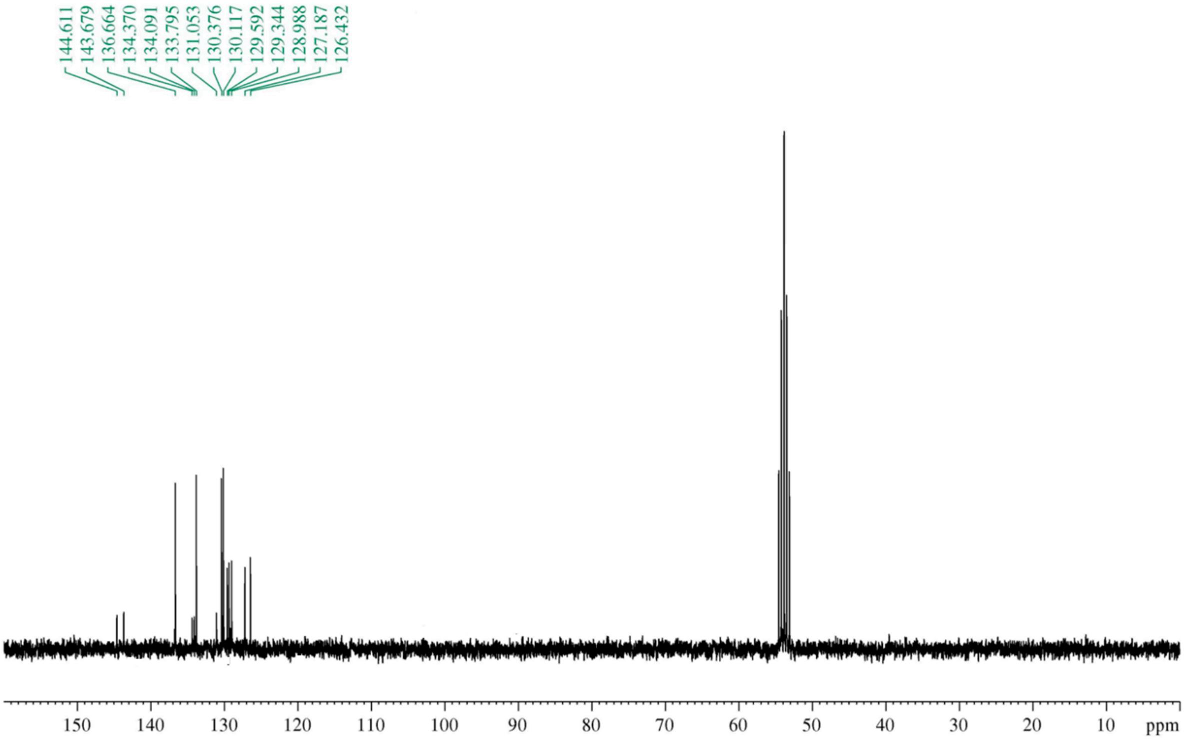

Figure S31: $^{13}C\{^{1}H\}$ NMR spectrum of compound **12**, CD_2Cl_2, 75 MHz, 25 °C.

2.13 Synthesis of Compound 13

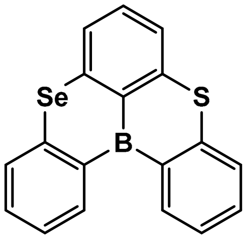

13

A solution of *n*-BuLi (1.05 eq., 1.35 mL, 2.5 M, 3.4 mmol) was slowly added to a suspension of compound **12** (1.00 eq., 1.50 g, 3.2 mmol) in anhydrous *m*-xylene (50 mL) at −30 °C. The suspension was heated to 50 ºC and stirred for 1 h. The reaction was cooled to −30 ºC, at which boron tribromide (1.50 eq., 1.20 g, 0.50 mL, 4.8 mmol) was slowly added. The mixture was stirred at room temperature for 1 h. Hünig Base (diisopropylethyl amine, 2.50 eq., 1.10 g, 1.40 mL, 8.0 mmol) was added at 0 °C. The reaction mixture was stirred at 125 ºC for 12 h. The suspension was cooled to room temperature. Sodium acetate aqueous solution (1 M, 50 mL) was added and the mixture was extracted three times with ethyl acetate (3 × 50 mL). The organic phase was dried over $MgSO_4$, and the solvent was removed under vacuum. The residue was crystallized from toluene by layering with *n*-pentane to obtain compound **13** in a yield of 75 % as a yellow crystalline material.

^{1}H NMR (300 MHz, $CDCl_3$): δ (ppm) = 8.19 (dd, ^{3}J= 7.70 Hz, ^{3}J= 1.52 Hz, 1H, aryl-CH), 8.13 (dd, ^{3}J= 7.50 Hz, ^{3}J= 1.80 Hz, 1H, aryl-CH), 7.79 (dq, ^{3}J= 7.77 Hz, ^{3}J= 1.30 Hz, 1H, aryl-CH), 7.73 (dq, ^{3}J= 8.10 Hz, ^{3}J= 1.30 Hz, 1H, aryl-CH), 7.69 (dd, ^{3}J= 7.77 Hz, ^{3}J= 1.04 Hz, 1H, aryl-CH), 7.63-7.55 (m, 2H, aryl-CH), 7.51-7.39 (m, 4H, aryl-CH).

^{11}B{^{1}H} NMR (96 MHz, $CDCl_3$): δ (ppm) = 48.2 (s, $\omega_{½}$ = 977 Hz).

^{13}C{^{1}H} NMR (75 MHz, $CDCl_3$): δ (ppm) = 143.87 (aryl-C), 143.36 (aryl-C), 142.04 (aryl-C), 141.96 (aryl-C), 140.03 (aryl-CH), 138.89 (aryl-CH), 130.78 (aryl-CH), 130.61 (aryl-CH), 129.78 (aryl-CH), 127.75 (aryl-CH), 125.45 (aryl-CH), 125.18 (aryl-CH), 124.82 (aryl-CH), 123.93 (aryl-CH), 122.26 (aryl-CH). Not observed B-*C*.

^{77}Se{^{1}H} NMR (95 MHz, $CDCl_3$): δ (ppm) = 409.2.

Elemental analysis. Calculated for $C_{18}H_{11}BSSe$: C, 61.93; H, 3.18; Found: C, 61.85; H, 3.21.

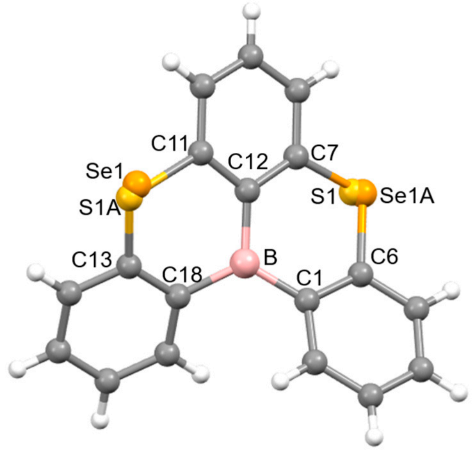

Figure S32: Molecular structure of compound **13**. Only the *P*-enantiomer of the asymmetric unit is shown. The occupancy at the sulfur and selenium positions were both found to be close to 0.5 by the refinement of a free variable. Selected bond distances and bond angles are reported in Å or degree (°), respectively. Se(1A)-C(7) 1.833(4), Se(1A)-C(6) 1.859(4), C(1)-C(6) 1.408(4), C(1)-B(1) 1.547(4), C(12)-C(7) 1.414(5), C(12)-B(1) 1.541(4), C(11)-Se(1) 1.795(4), C(13)-C(18) 1.3900, C(13)-Se(1) 1.906(2), C(18)-B(1) 1.581(4), C(1)-C(6)-S(1A) 116.9(3), C(6)-C(1)-B(1) 122.6(3), C(12)-C(7)-Se(1A) 124.7(2), C(12)-C(11)-Se(1) 123.4(3), C(7)-C(12)-B(1) 122.0(3), C(11)-C(12)-C(7) 115.3(3), C(18)-C(13)-Se(1) 123.58(13), C(13)-C(18)-B(1) 120.05(18), C(12)-B(1)-C(1) 118.5(3), C(12)-B(1)-C(18) 118.2(3), C(1)-B(1)-C(18) 123.2(3), C(11)-Se(1)-C(13) 100.19(15).

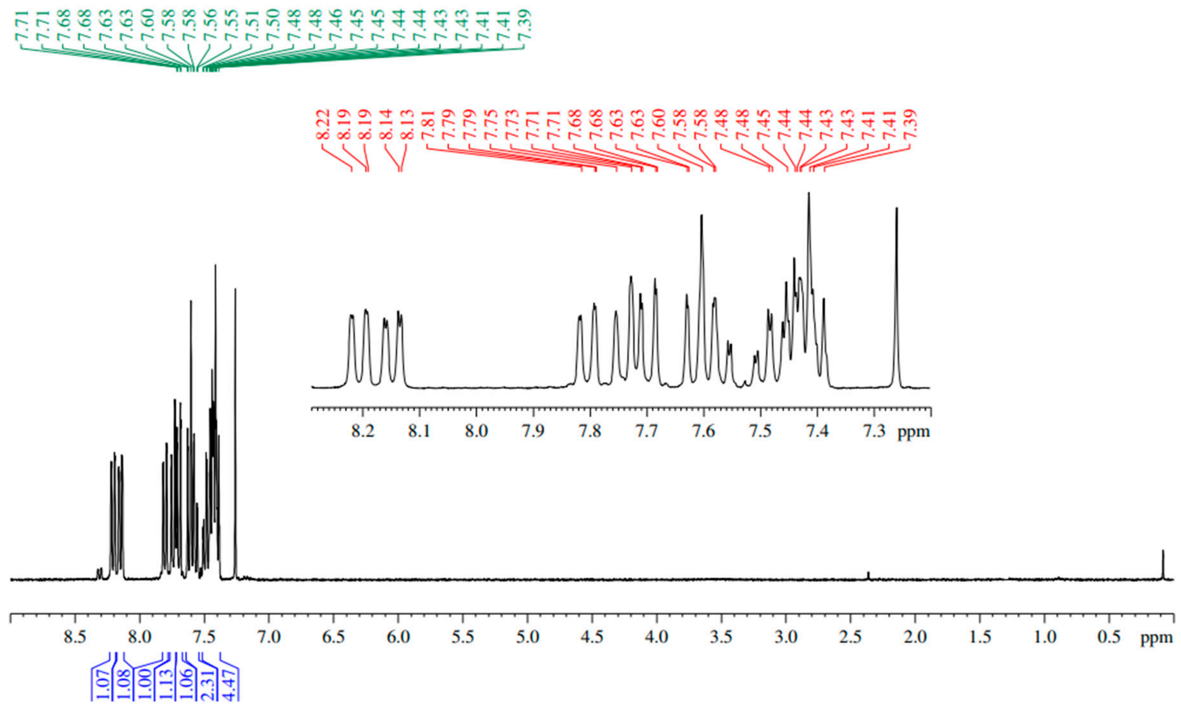

Figure S33: ¹H NMR spectrum of compound **13**, $CDCl_3$, 300 MHz, 25 °C.

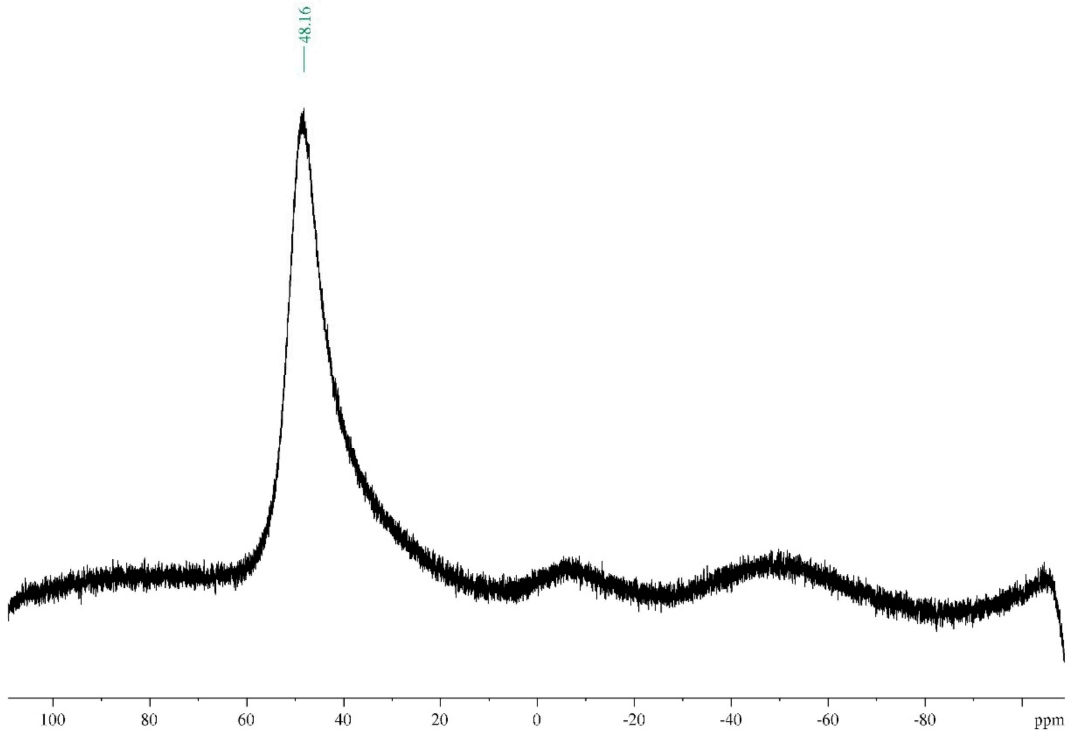

Figure S34: ^{11}B NMR spectrum of compound **13**, $CDCl_3$, 96 MHz, 25 °C.

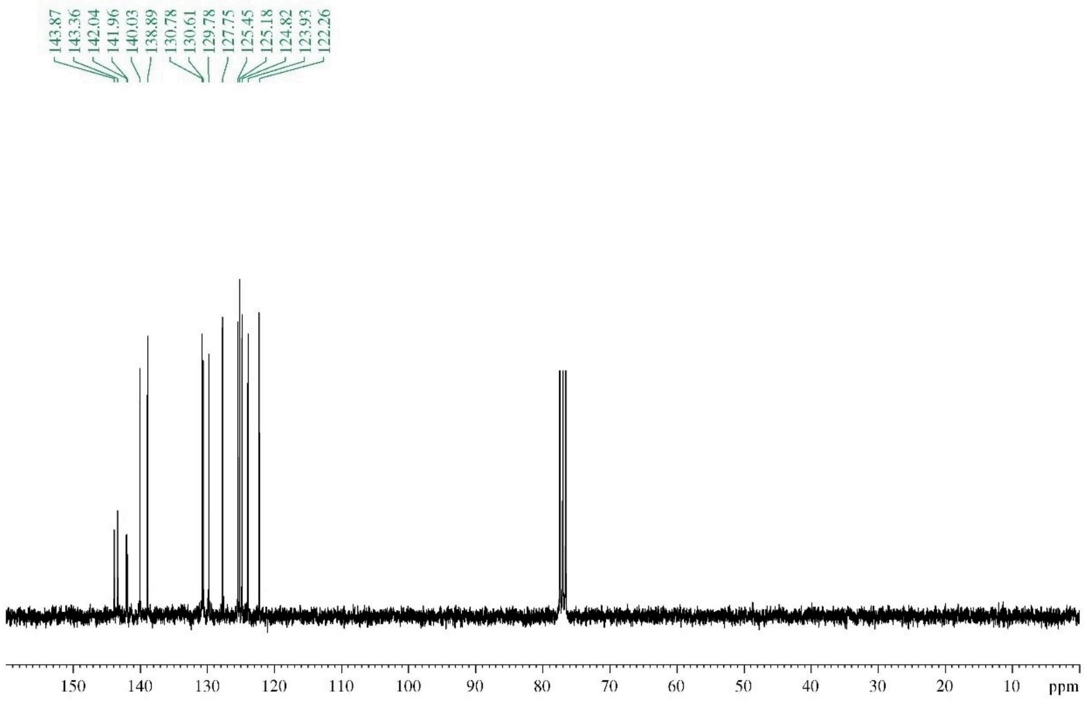

Figure S35: $^{13}C\{^1H\}$ NMR spectrum of compound **13**, $CDCl_3$, 75 MHz, 25 °C.

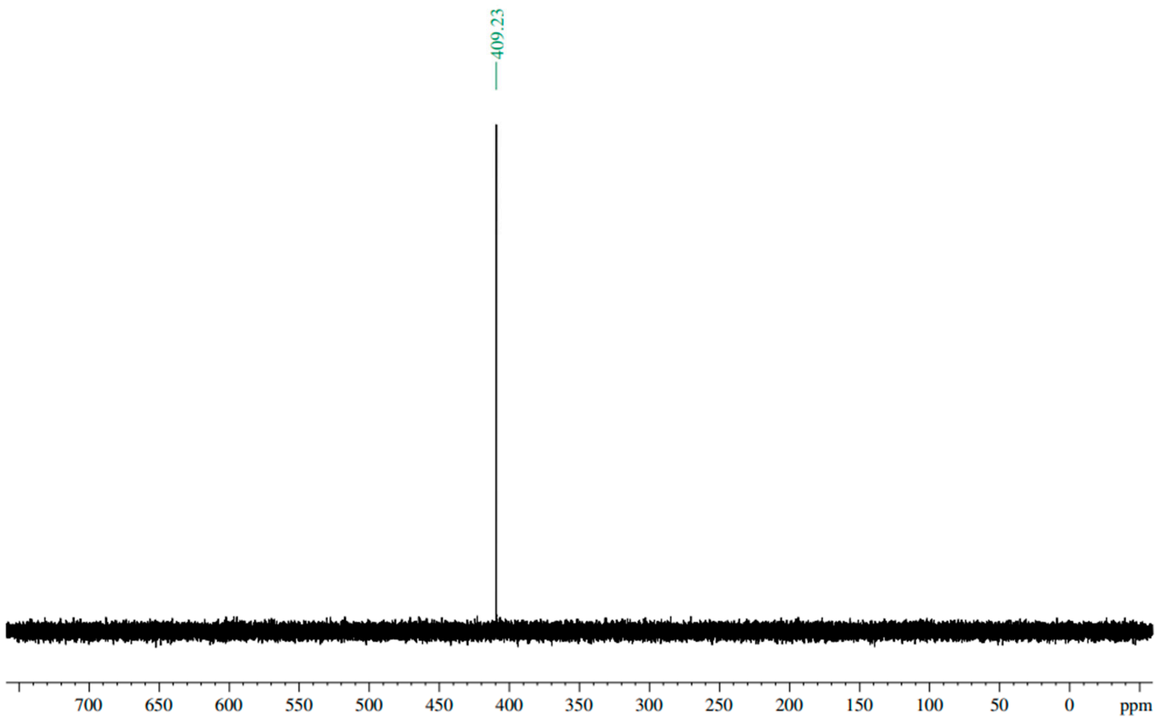

Figure S36: ^{77}Se{^{1}H} NMR spectrum of compound **13**, $CDCl_3$, 95 MHz, 25 °C.

2.14 Synthesis of Compound 14

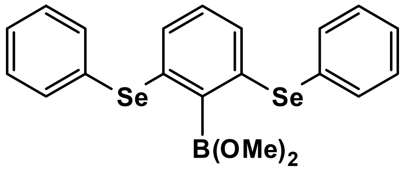

14

A solution of *n*-BuLi (1.00 eq., 1.30 mL, 2.5 M, 3.2 mmol) was slowly added to a suspension of compound **12** (1.00 eq., 1.50 g, 3.2 mmol) in anhydrous *m*-xylene (50 mL) at −30 °C. The suspension was heated to 50 ºC and stirred for 1 h. The reaction was cooled to −30 ºC, at which trimethoxy borane (1.56 eq., 0.52 g, 0.56 mL, 5.0 mmol) was slowly added. The mixture was stirred at room temperature for 1 h and before all volatile components were removed *in vacuo*. The residue was suspended in *n*-hexane (200 mL) stirred overnight. Filtration and removal of the solvent afforded compound **14** in a yield of 94 % as a slightly yellowish powder.

^{1}H NMR (300 MHz, CDCl$_3$): δ (ppm) = 7.43-7.40 (m, 4H, aryl-CH), 7.36 (d, ^3J= 7,7 Hz, 2H, aryl-CH), 6.91-6.88 (m, 6H, aryl-CH), 6.70 (tr, ^3J= 7,7 Hz, 1H, aryl-CH), 3.51 (s, 6H, OMe).

^{11}B{^{1}H} NMR (96 MHz, CDCl$_3$): δ (ppm) = 28.0 (s, ω½ = 565 Hz).

^{13}C{^{1}H} NMR (75 MHz, CDCl$_3$): δ (ppm) = 134.37 (aryl-CH), 134.20 (aryl-C), 132.80 (aryl-CH), 132.40 (aryl-C), 130.45 (aryl-CH), 129.50 (aryl-CH), 127.28 (aryl-CH), 52.21 (O-C). Not observed B-*C*.

Elemental analysis. Calculated for $C_{18}H_{11}BSSe$: C, 61.93; H, 3.18; Found: C, 61.85; H, 3.21.

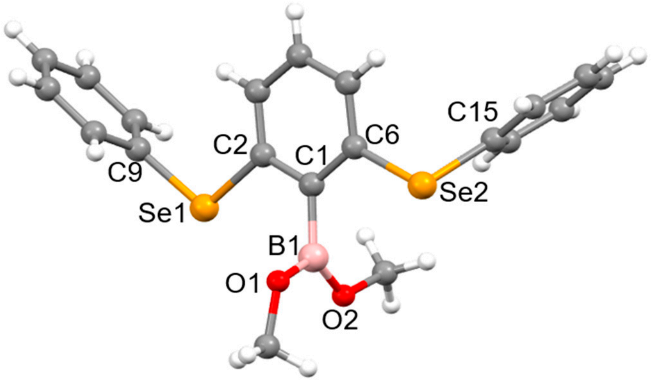

Figure S37: Molecular structure of compound **14**. Hydrogen atoms are omitted for clarity. Bond distances and bond angles are reported in Å or degree (°), respectively. Se(1)-C(9) 1.9165(18), Se(1)-C(2) 1.9312(16), Se(2)-C(15) 1.9251(18), Se(2)-C(6) 1.9306(17), O(1)-B(1) 1.356(2), O(1)-C(8) 1.439(2), O(2)-B(1) 1.346(2), C(1)-C(6) 1.404(2), C(1)-C(2) 1.409(2), C(1)-B(1) 1.589(2), C(6)-C(1)-C(2) 117.11(15), C(6)-C(1)-B(1) 122.17(15), C(2)-C(1)-B(1) 120.49(14), C(1)-C(2)-Se(1) 116.29(12), O(2)-B(1)-O(1) 119.80(16), O(2)-B(1)-C(1) 125.31(15), O(1)-B(1)-C(1) 114.81(15).

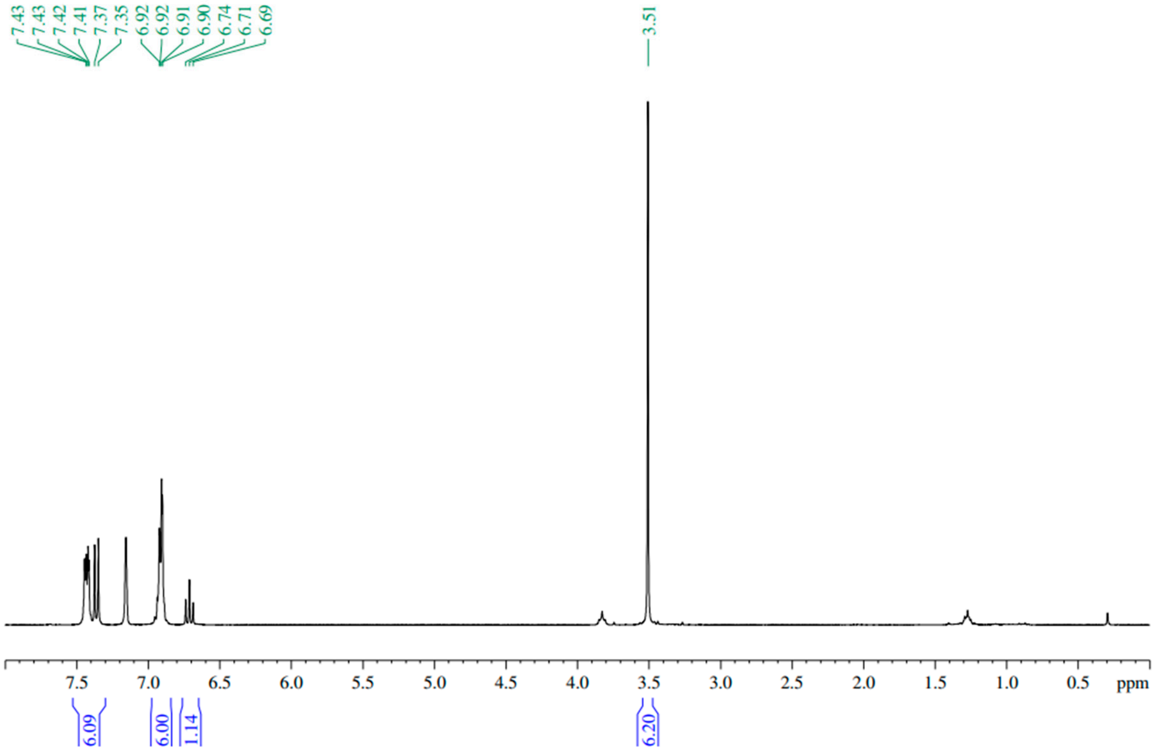

Figure S38: ^{1}H NMR spectrum of compound **14**, C_6D_6, 300 MHz, 25 °C.

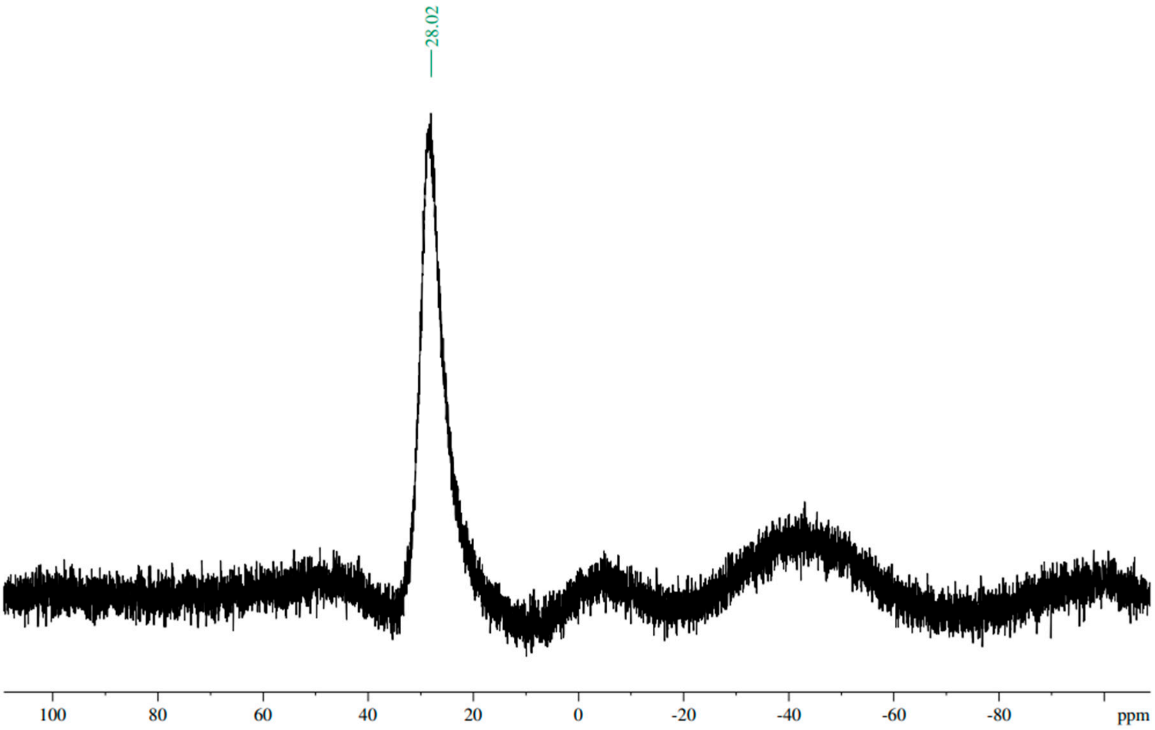

Figure S39: ^{11}B NMR spectrum of compound **14**, C_6D_6, 96 MHz, 25 °C.

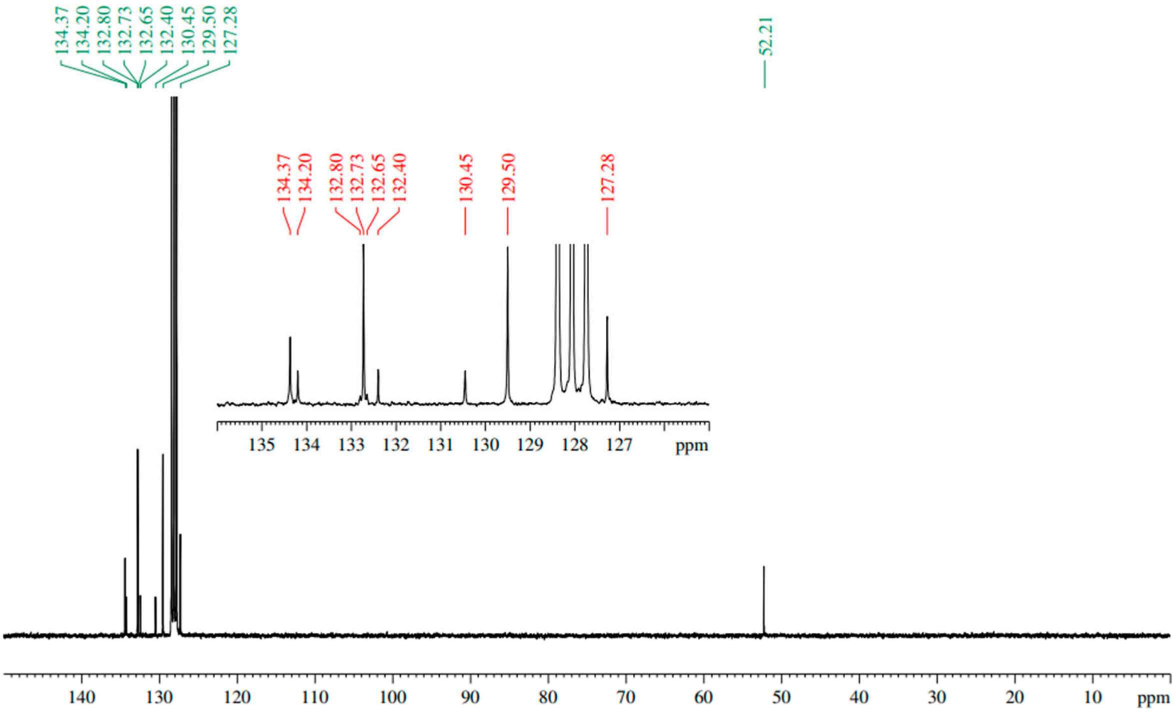

Figure S40: $^{13}C\{^1H\}$ NMR spectrum of compound **14**, C_6D_6, 75 MHz, 25 °C.

2.15 Alternative synthesis of compound 8

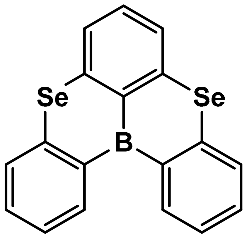

8

A solution of compound **14** (1.0 eq., 1.00 g, 2.2 mmol) in anhydrous *m*-xylene (30 mL) was cooled to −30 ºC, at which boron tribromide (2.20 eq., 1.20 g, 0.50 mL, 4.8 mmol) was slowly added. The mixture was stirred at room temperature for 1 h. Hünig Base (diisopropylethyl amine, 3.00 eq., 0.84 g, 1.11 mL, 6.5 mmol) was then added at 0 °C. The reaction mixture was stirred at 125 ºC for 12 h. The suspension was cooled to room temperature. Sodium acetate aqueous solution (1 M, 50 mL) was added and the mixture was extracted three times with ethyl acetate (3 × 50 mL). The organic phase was dried over $MgSO_4$, and the solvent was removed under vacuum. The residue was crystallized from toluene by layering with *n*-pentane to obtain compound **8** in a yield of 70 % as a yellow crystalline material.

The analytical data of this material were in accordance with those mentioned above.

3. X-Ray Crystallography

Data collections were performed by mounting single crystals on glass fibers or MiTeGen mounts in perfluorinated oil. Diffractometers used for intensity measurements (at 100 K) were Oxford Diffraction Xcalibur E with Mo K_α radiation or Rigaku XtaLab Synergy S Single Source with either Mo K_α or Cu K_α micro source. Absorption correction was applied based on multi-scan methods. Data reduction was performed using the program CrysalisPro.[1] The structures were solved with SHELXT-18/2 [2] and refined anisotropically on F^2 using the program SHELXL-18/3.[3]

Compound	Compound **3**	Compound **4**
CCDC entry code	2394813	2394817
Empirical formula	$C_{18}H_{13}IS_2$	$C_{18}H_{11}BS_2$
Formula weight	420.30	302.20
Temperature	100(2) K	100(2) K
Wavelength	1.54184	0.71073 Å
Crystal system	monoclinic	Orthorhombic
Space group	$P2_1/c$	*Pbca*
a	15.7878(2) Å	16.5350(2) Å
b	13.0646(2) Å	7.68020(10) Å
c	7.76100(10) Å	21.5326(2) Å
α	90°	90°
β	98.1820(10)°	90°
γ	90°	90°
Volume	1584.50(4) Å^3	2734.47(5) Å^3
Z	4	8
Density (calculated)	1.762 Mg/m^3	1.468 Mg/m^3
Absorption coefficient	18.243 mm^{-1}	0.376 mm^{-1}
F(000)	824	1248
Crystal size	0.110 x 0.040 x 0.040 mm^3	0.520 x 0.290 x 0.160 mm^3
θ-range for data collection	2.828 to 77.375°	2.257 to 44.918°
Reflections collected	64213	158147
Independent reflections	3360 [R(int) = 0.0345]	11217 [R(int) = 0.0816]
Goodness-of-fit on F^2	1.080	1.051
R_1 $[I > 2\sigma(I)]$	0.0164	0.0388
wR_2	0.0400	0.0915

Compound	Compound **7**	Compound **8**
CCDC entry code	2394812	2394816
Empirical formula	$C_{18}H_{13}ISe_2$	$C_{18}H_{11}BSe_2$
Formula weight	514.10	396.00
Temperature	100(2) K	105(2) K
Wavelength	1.54184 Å	1.54184 Å
Crystal system	Monoclinic	Orthorhombic
Space group	$P2_1/c$	$P2_12_12_1$
a	15.7206(3) Å	7.72054(4) Å
b	13.4033(2) Å	18.76858(9) Å
c	7.83020(10) Å	19.61219(10) Å
α	90°	90°
β	98.947(2)°	90°
γ	90°	90°
Volume	1629.81(5) $Å^3$	2841.88(2) $Å^3$
Z	4	8
Density (calculated)	2.095 Mg/m^3	1.851 Mg/m^3
Absorption coefficient	20.451 mm^{-1}	6.381 mm^{-1}
F(000)	968	1536
Crystal size	0.090 x 0.090 x 0.050 mm^3	0.260 x 0.220 x 0.120 mm^3
θ-range for data collection	2.845 to 76.163°	3.259 to 80.313°
Reflections collected	35107	125610
Independent reflections	3360 [R(int) = 0.0434]	6085 [R(int) = 0.0738]
Goodness-of-fit on F^2	1.217	1.106
R_1 $[I > 2\sigma(I)]$	0.0359	0.0213
wR_2	0.089	0.0591

Compound	Compound **12**	Compound **13**
CCDC entry code	2394814	2394815
Empirical formula	$C_{18}H_{13}ISSe$	$C_{18}H_{11}BSSe$
Formula weight	467.20	349.10
Temperature	100(2) K	100(2) K
Wavelength	0.71073 Å	0.71073 Å
Crystal system	Monoclinic	Orthorhombic
Space group	$P2_1/c$	$P2_12_12_1$
a	15.8213(2) Å	7.61150(10) Å
b	13.23350(10) Å	18.7823(2) Å
c	7.75580(10) Å	19.6049(2) Å
α	90°	90°
β	98.5170(10)°	90°
γ	90°	90°
Volume	1605.93(3) $Å^3$	2802.75(6) $Å^3$
Z	4	8
Density (calculated)	1.932 Mg/m^3	1.655 Mg/m^3
Absorption coefficient	4.381 mm^{-1}	2.815 mm^{-1}
F(000)	896	1392
Crystal size	0.270 x 0.160 x 0.110 mm^3	0.450 x 0.320 x 0.310 mm^3
θ-range for data collection	2.603 to 37.035°	2.078 to 32.031°
Reflections collected	78866	90877
Independent reflections	8174 [R(int) = 0.0345]	9684 [R(int) = 0.0420]
Goodness-of-fit on F^2	1.139	1.056
R_1 [$I > 2\sigma(I)$]	0.0349	0.0439
wR_2	0.0717	0.0842

Compound	Compound **14**
CCDC entry code	2394811
Empirical formula	$C_{20}H_{19}BO_2Se_2$
Formula weight	460.08
Temperature	100(2) K
Wavelength	0.71073 Å
Crystal system	Triclinic
Space group	*P*-1
a	6.91140(10) Å
b	9.38480(10) Å
c	15.1930(2) Å
α	105.4210(10)°
β	90.9120(10)°
γ	90.8370(10)°
Volume	949.67(2) Å^3
Z	2
Density (calculated)	1.609 Mg/m^3
Absorption coefficient	3.903 mm^{-1}
F(000)	456
Crystal size	0.210 x 0.090 x 0.050 mm^3
θ-range for data collection	2.252 to 32.917°
Reflections collected	30382
Independent reflections	6520 [R(int) = 0.0383]
Goodness-of-fit on F^2	1.030
R_1 [$I > 2\sigma(I)$]	0.0477
wR_2	0.0708

[1] Rigaku Oxford Diffraction, *"CrysAlisPRO Softw. Syst. version 1.171.39.46"* **2018**, Rigaku Corporation, Oxford, UK. (Ed.).
[2] G. M. Sheldrick, *Acta Cryst.* **2015**, *A71*, 3–8.
[3] G. M. Sheldrick, *Acta Crystallogr., Sect. A Found. Crystallogr.* **2008**, *64*, 112-122.
